# The Handicap Principle: how an erroneous hypothesis became a scientific principle

**DOI:** 10.1111/brv.12563

**Published:** 2019-10-23

**Authors:** Dustin J. Penn, Szabolcs Számadó

**Affiliations:** ^1^ Konrad Lorenz Institute of Ethology University of Veterinary Medicine Vienna Austria; ^2^ Department of Sociology and Communication Budapest University of Technology and Economics Budapest Hungary; ^3^ CSS‐ RECENS MTA Centre for Social Sciences Budapest Hungary; ^4^ Evolutionary Systems Research Group MTA Centre for Ecological Research Tihany Hungary

**Keywords:** animal communication, handicap hypothesis, costly signalling theory, honest signalling, sexual selection, strategic handicap model, conspicuous consumption, scientific bandwagon, confirmation bias, affirming the consequent

## Abstract

The most widely cited explanation for the evolution of reliable signals is Zahavi's so‐called Handicap Principle, which proposes that signals are honest because they are costly to produce. Here we provide a critical review of the Handicap Principle and its theoretical development. We explain why this idea is erroneous, and how it nevertheless became widely accepted as the leading explanation for honest signalling. In 1975, Zahavi proposed that elaborate secondary sexual characters impose ‘handicaps’ on male survival, not due to inadvertent signalling trade‐offs, but as a mechanism that functions to demonstrate males' genetic quality to potential mates. His handicap hypothesis received many criticisms, and in response, Zahavi clarified his hypothesis and explained that it assumes that signals are wasteful as well as costly, and that they evolve because wastefulness enforces honesty. He proposed that signals evolve under ‘signal selection’, a non‐Darwinian type of selection that favours waste rather than efficiency. He maintained that the handicap hypothesis provides a general principle to explain the evolution of all types of signalling systems, i.e. the Handicap Principle. In 1977, Zahavi proposed a second hypothesis for honest signalling, which received many different labels and interpretations, although it was assumed to be another example of handicap signalling. In 1990, Grafen published models that he claimed vindicated Zahavi's Handicap Principle. His conclusions were widely accepted and the Handicap Principle subsequently became the dominant paradigm for explaining the evolution of honest signalling in the biological and social sciences. Researchers have subsequently focused on testing predications of the Handicap Principle, such as measuring the absolute costs of honest signals (and using energetic and other proximate costs as proxies for fitness), but very few have attempted to test Grafen's models. We show that Grafen's models do not support the handicap hypothesis, although they do support Zahavi's second hypothesis, which proposes that males adjust their investment into the expression of their sexual signals according to their condition and ability to bear the costs (and risks to their survival). Rather than being wasteful over‐investments, honest signals evolve in this scenario because selection favours efficient and optimal investment into signal expression and minimizes signalling costs. This idea is very different from the handicap hypothesis, but it has been widely misinterpreted and equated to the Handicap Principle. Theoretical studies have since shown that signalling costs paid at the equilibrium are neither sufficient nor necessary to maintain signal honesty, and that honesty can evolve through differential benefits, as well as differential costs. There have been increasing criticisms of the Handicap Principle, but they have focused on the limitations of Grafen's model and overlooked the fact that it is not a handicap model. This model is better understood within a Darwinian framework of adaptive signalling trade‐offs, without the added burden and confusing logic of the Handicap Principle. There is no theoretical or empirical support for the Handicap Principle and the time is long overdue to usher this idea into an ‘honorable retirement’.

## I. INTRODUCTION

*[The Handicap Principle is] one of the most enduring and well known of all theories in animal behavior and behavioral ecology* … (Higham, 2014, p. 8)

Explaining the evolution of honest signals has been a major theoretical challenge in animal communication (Maynard Smith & Harper, 2003; Searcy & Nowicki, 2005). Honest or reliable signalling is particularly puzzling when there are conflicts of interest between senders *versus* receivers, and deception is feasible and potentially beneficial. For example, males of some species develop conspicuous secondary sexual traits that provide reliable indicators of their condition, health and social status, raising the question: what prevents poor‐quality males from cheating? There are many examples of reliable signals, but deception is also common and is expected to drive coevolutionary ‘arms races’ between signallers and receivers (Dawkins & Krebs, 1979). Understanding deceptive signals and our own species' vulnerability to misinformation, disinformation, and propaganda in the modern world (Akerlof & Shiller, 2015; Kopp, Korb & Mills, 2018) might even benefit from biological perspectives on human communication.

The most popular explanation for the evolution of honest signals is Amotz Zahavi's (1975) Handicap Principle. He proposed several versions and there are many different interpretations of this concept, although the most generic one proposes that signals must be costly to be reliable. For example, Zahavi argued, ‘…in order to be effective, signals have to be reliable; in order to be reliable, signals have to be costly’ (Zahavi & Zahavi, 1997, p. XIV). Zahavi's original goal was to explain the evolution of costly and conspicuous secondary sexual signals, such as the colourful plumage of peacocks. He likened such sexual displays to ‘handicaps’ because they potentially reduce survival, and he argued that costly signals are beneficial because they demonstrate a male's quality reliably to potential mates and rivals. If signals do not have extra costs on survival, he argued, they would be easily cheated, and eventually become ignored. Zahavi maintained that his proposal is not merely a hypothesis, but rather a general scientific principle, which he called the *Handicap Principle*, that explains the evolution of all honest signals and all types of biological signalling systems (Zahavi, 1975, 1981, 1987; Zahavi & Zahavi, 1997).

**Figure 1:**
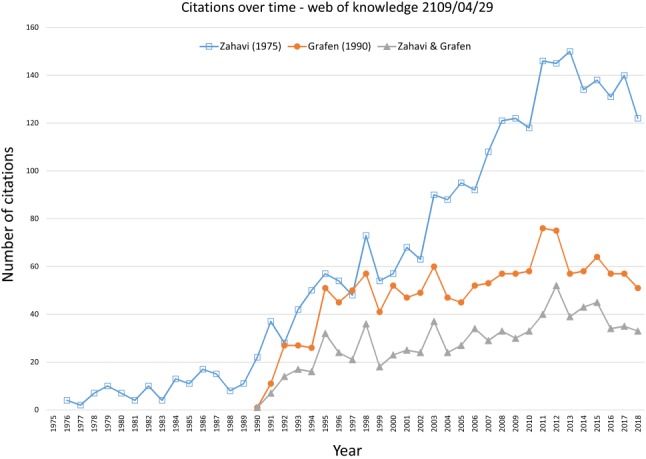
Citations of Zahavi's Handicap Principle before and after Grafen's (1990a) strategic choice ‘handicap’ model, and the number of studies that cite both authors for the Handicap Principle.

The Handicap Principle is the most widely accepted explanation for explaining honest signalling in the biological sciences (Maynard Smith & Harper, 2003; Searcy & Nowicki, 2005; Bradbury & Vehrencamp, 2011) (Fig. 1). It inspired an explosion of research on sexual selection and animal communication (Andersson, 1994; Johnstone, 1995). The Handicap Principle was preceded by Veblen's (1899) conspicuous consumption and Spence's (1973) job market signalling model in the social sciences. These ideas are often merged under the rubric of ‘costly signalling theory’ and proposed to explain many puzzling and seemingly wasteful human behaviours, including generosity, inefficient foraging, risk‐taking, over‐consumption of resources, monumental architecture, and religious rituals (Boone, 1998; Bliege Bird, Smith & Bird, 2001; Hawkes & Bliege Bird, 2002; McAndrew, 2019). The Handicap Principle is featured in most textbooks on animal behaviour and animal communication, and it is promoted by many popular books on human behaviour and evolution. As one reviewer pointed out, the Handicap Principle has become ‘one of the most enduring and well known of all theories in animal behaviour and behavioural ecology … and has been adopted by other fields, such as evolutionary psychology and human evolution’ (Higham, 2014, p. 8). It has become a scientific paradigm with its own particular terminology and theoretical framework to model, test, and interpret hypotheses for the evolution of costly and honest signals.

Zahavi's Handicap Principle was initially very controversial, but it gained widespread acceptance following Grafen's (1990a) influential paper, *Biological Signals as Handicaps* (Fig. 1). Grafen provided a model to show how honest signals of male quality can evolve through sexual selection if low‐quality males pay greater marginal fitness costs for signalling compared to high‐quality males. He concluded that his results provided a general explanation for honest signalling that vindicates Zahavi's Handicap Principle. As we will show, however, this model was misinterpreted. It does not provide a general explanation for honest signals, and it does not support the Handicap Principle. On the contrary, it offers a Darwinian *alternative* to the Handicap Principle for explaining the evolution of honest signals. The problem is not that Grafen's model is a ‘watered‐down version’ of the Handicap Principle, as one reviewer described it (Cronin, 1991, p. 197), but rather that it is based on a completely different logic. Yet, researchers continue publishing theoretical and empirical papers that confound Grafen's model with the Handicap Principle, and some continue to claim to provide support for Zahavi's Handicap Principle (e.g. see Számadó & Penn, 2015, 2018).

The Handicap Principle has received criticisms since the publication of Grafen's (1990a,*b*) papers in biology (Getty, 2006; Grose, 2011; Számadó, 2011; Higham, 2014) and anthropology (Barker *et al*., 2019; Stibbard‐Hawkes, 2019). However, no reviews have summarized the theoretical development of the Handicap Principle and none have addressed the full extent of the theoretical problems with Zahavi's proposals or misinterpretations of Grafen's models. The number of empirical studies that contradict the assumptions and predictions of the Handicap Principle are increasing steadily. Many studies refute the assumptions that signals are usually honest (Backwell *et al*., 2000; Christy & Rittschof, 2011; Brown, Garwood & Williamson, 2012) and very costly to produce (Borgia, 1993, 1996; McCarty, 1996; Moreno‐Rueda, 2007; McCullough & Emlen, 2013; Askew, 2014; Thavarajah *et al*., 2016; Guimarães *et al*., 2017). These are only a few of the many studies that report inconsistent findings, and moreover there is no evidence that signals are honest *because* they are costly. These anomalies have largely been ignored and theoreticians have offered no way to resolve these problems. Thus, a comprehensive re‐evaluation of the entire Handicap Paradigm (and costly signalling theory) is needed.

Here, we critically evaluate Zahavi's Handicap Principle, and provide a comprehensive overview of its theoretical development and main problems. We explain why this idea is illogical and contrary to Darwinian principles, and why it is not supported by Grafen's (or any other) theoretical models, contrary to what has been widely assumed. We also explain why Grafen's models, although logical and Darwinian, do not provide a general explanation for honest signalling. More specifically, we address the following issues. First, we examine ambiguities in terminology that have caused conceptual confusion, and we clarify how we will use the terms *handicap*, *handicap hypothesis* and *handicap principle* (Section II). Second, we evaluate Zahavi's (1975) original proposal and his subsequent attempts to clarify his Handicap Principle (Zahavi, 1977a, 1981, 1987; Zahavi & Zahavi, 1997) (Sections III and IV). Third, we examine another hypothesis that Zahavi (1977b) proposed to explain reliable signals, which is logical and consistent with evolutionary biology, but widely misinterpreted (Sections V and VI). Fourth, we examine Grafen's (1990a) *strategic choice signalling* model, and show how it provided theoretical support for Zahavi's (1977b) second hypothesis, but not the Handicap Principle (Section VII). We explain how the arguments used to justify this claim are based on several misinterpretations. Finally, we propose several explanations for why Grafen's handicap conclusions carried so much weight and became widely accepted (Section VIII). We hope that by clarifying the differences between the Handicap Principle and Grafen's models (and other hypotheses that have been mistaken as handicap models), we can reject this erroneous concept and put an end to this long debate.

## II. WHAT IS THE HANDICAP PRINCIPLE?

*One cause of confusion has been that different authors have used the same term with different meanings, and different terms with the same meaning*. (Maynard Smith & Harper, 2003, Preface)

The literature on the Handicap Principle is plagued with semantic confusion, and there is little agreement over how to define, model or test this idea (Számadó & Penn, 2015, 2018; Számadó, Czégel & Zachar, 2019). Different authors use terms differently, and the *same* author often uses the same term for very different ideas – and different terms for the same concept – in the same paper (although Maynard Smith recognized this problem, he also contributed to the confusion, as we later show). This criticism does not mean that the disagreements over the Handicap Principle are merely quibbles over words, but rather that ambiguous terms must be clarified in order to address the actual theoretical issues. The semantic confusion began with Zahavi's papers, which are like works of art: there are many interpretations about what he apparently *meant* to say, and different interpretations are treated as if they are equally valid. In this section, we show how the terms ‘handicap’, ‘handicap hypothesis’ and ‘handicap principle’ have been used to refer to very different ideas, and we clarify how we will use these terms here to avoid confusion.

The term ‘handicap’ has several different meanings in the literature, although it is mainly used to refer to the following: (*i*) Zahavi (1975) first used ‘handicap’ to refer to male secondary sexual signals that attract females, but that also reduce survival (or so he assumed). Such viability costs or signalling trade‐offs are not as well documented as generally assumed (Kotiaho, 2001), even though they have been expected since Darwin. Moreover, as West‐Eberhard (1979) pointed out, ‘if one accepts the premise that every character costs something to produce or maintain, the trade off of taking on a handicap because of some overriding benefit in another is common place in evolution’ (p. 227). From this perspective, every trait with a viability cost becomes a handicap; (*ii*) Zahavi used the term ‘handicap’ to refer to hypothetical signals that are *wasteful* or costlier than they need to be (and have viability costs); and (*iii*) He also used the term ‘handicap’ to refer to hypothetical signals that are honest and evolve *because* they are costly to produce (Sections III and IV). We refer to such hypothetical signals as *Zahavian handicaps*. The term ‘handicap’ has additional meanings depending upon the particular interpretation of the Handicap Principle, and therefore, we avoid this ambiguous term (except to provide quotes).

The term ‘handicap principle’ also has several different meanings, and we distinguish two very different usages. (*i*) This term has often been used as a synonym for the handicap hypothesis, which should be avoided as it confuses the important distinction between a hypothesis *versus* a scientific principle; and (*ii*) It is often used to refer to the claim that the handicap hypothesis provides a general principle to explain the evolution of all types of signalling systems (or at least honest signals) (Section IV.4). Here, we use the term ‘Handicap Principle’ in this latter strict sense, and in upper case for clarification (and lower case only for quoting others).

The term ‘handicap hypothesis’ is used to refer to a wide variety of different, albeit related ideas and models, and we restrict this term to Zahavi's (1975, 1977b, 1981, 1987) proposal that signals must be costly to produce and reduce survival in order to be reliable (Sections III and IV). This definition is the broad, generic version and there are other versions, such as the idea that costly signals are honest indicators of quality (‘quality handicap’). Zahavi also advocated a weaker version, and suggested that signals are wasteful, and that their wastefulness makes them reliable (Section IV.2). This hypothesis – regardless of how it is labelled – is incomplete, however, as it does not specify *how* signal costs (or wastefulness) can maintain reliability at proximate or ultimate levels. It still requires providing a testable mechanism that explains how it works; how signal costs enforce or maintain honesty. Several such mechanisms have been proposed, including the fixed (or epistatic) handicap, the condition‐dependent handicap, the revealing handicap, and the strategic handicap models (Sections V and VI). These proposals are labelled and classified as ‘handicap models’, however, we consider them to be *putative* handicap models – until they can be shown to explain how signal costs (or wastefulness) are necessary to maintain the evolution of signal honesty. Otherwise, we consider them to be pseudo‐handicap models. Maynard Smith proposed that signals can be honest due to unfakeable constraints, which he initially labelled as ‘revealing handicaps’ (Maynard Smith, 1985), but later reclassified and relabelled as ‘index signals’ (Maynard Smith & Harper, 1995, 2003) (Section VI.1). Honest signals can also be explained by costly punishment, but contrary to what is often assumed, this is not a handicap model (Fraser, 2012; Webster, Ligon & Leighton, 2018). Similarly, we show that Grafen's (1990a) so‐called *strategic handicap* model is not a model of the handicap hypothesis nor a general principle.

The terms ‘handicap principle’ and ‘handicap hypothesis’ are widely used to refer to both Zahavi's (1975) Handicap Principle and Grafen's (1990a) strategic choice model, and both authors are often cited (see Fig. 1). This usage confuses the important differences between these proposals, and contributes to the misconception that the Handicap Principe has been validated. In the next two sections, we summarize Zahavi's proposals for his handicap hypothesis and Handicap Principle, and explain why they should be rejected.

## III. ZAHAVI'S HANDICAP HYPOTHESIS

*Many, if not all, sexual displays endanger their performers. Many of them seem to be designed specifically for that purpose*. (Zahavi, 1975, p. 211)

*My aim is to call attention to the possibility that the value of many characters may reside in their action as testing devices*… (Zahavi, 1975, p. 209)

In Zahavi's (1975) classic paper, *Mate selection – a selection for a handicap*, he aimed to propose an explanation for the evolution of conspicuous secondary sexual characters, such as the colourful plumage of peacocks. His paper sparked much interest in Darwin's sexual selection, which had been long ignored. Here we summarize the theoretical problem and Zahavi's first proposed solution, and explain why his arguments can be rejected.

### (1) The problem: costly secondary sexual signals

Darwin (1871) struggled to explain the evolution of secondary sexual characters because he did not see how natural selection could favour traits that are ‘injurious’ to survival. He was convinced that such traits must have a function, and he proposed that they evolve by enhancing mating and reproductive success, either by intimidating rivals or by attracting females. The beautiful feathers of some birds, such as the male Argus pheasant (*Argusianus argus*), are only displayed during courtship, and therefore, he argued that their only function must be to charm females. Darwin realized that natural selection is more than a struggle for survival, and that any traits that provide a reproductive advantage for one individual over another of the same sex will result in *sexual selection*. He proposed that sexual selection could explain the extraordinary variation of secondary sexual characters, including those that have been ‘carried to a wonderful extreme.’ He emphasized that sexual selection acts in a less rigorous manner than natural selection, because, rather than facing death, less successful males ‘merely’ fail to obtain a mate, they mate later in the season, or they obtain less vigorous females. He argued that ‘natural selection will determine that such characters shall not be acquired by the victorious males if they would be highly injurious to them, either by expending too much of their vital powers or by exposing them to any great danger’ (p. 257). The most extreme sexual characters, he argued, must have reproductive advantages that outweigh their disadvantages to survival in the long run. In other words, he recognized that male secondary sexual signals have fitness trade‐offs due to attracting the attention of predators as well as females, and that they can be favoured by selection only as long their reproductive benefits outweigh their negative effects on survival. It would take another century, however, until it was realized that individual (bodily) survival is only a proxy for fitness and the importance of reproductive success (genetic survival) would become appreciated.

Darwin's sexual selection theory lacked supporters for many years and mainly because he did not explain why females prefer to mate with ornamented males. He argued that a male's courtship display appeals to females' aesthetic tastes. His proposal provides a potential proximate explanation for female preferences (yet if ‘aesthetic taste’ is defined as showing a preference, as it often is, then this suggestion is merely a truism). Darwin's hypothesis did not provide a complete explanation, however, contrary to what some claim (Patricelli, Hebets & Mendelson, 2019), because it begs the question why females evolve such tastes. Darwin recognized that explaining the peacock requires explaining the peahen, but he took female preferences for granted. He did not provide a clear explanation for how they evolved, even if he came close to it. When he summarized his explanation for the evolution of secondary sexual characters through sexual selection, he argued that ‘the largest number of vigorous offspring’ are produced by females pairing with the most vigorous males, and also by males preferring the healthiest and most vigorous females (p. 249). Wallace (1895) argued that natural selection will make sure that females are ‘sensible’. If females seem to be attracted to beauty, then this is only because ‘as a rule’ the expression of secondary sexual traits is correlated with health and vigour – traits favoured by natural selection. Vigorous males will have the choice of the healthiest females, and together they will produce the ‘most numerous and healthy families’ (Wallace, 1895, p. 375). Wallace did not suggest how secondary sexual traits might indicate health and vigour, however.

Fisher (1915, 1930) realized that female preferences, like male secondary sexual traits, require an evolutionary explanation. He argued that the ‘tastes of organisms, like their organs and faculties, must be regarded as the product of evolutionary change, governed by the relative advantages which such tastes confer’ (Fisher, 1930, p. 151). He also proposed that females are attracted to the males with conspicuous secondary sexual traits because they provide a ‘rough index’ of their health and general vigour. His argument was forgotten and over‐shadowed by his other hypothesis. Fisher argued that if females evolve a preference for conspicuous male ornaments for any reason, then their offspring will inherit their preferences as well as their father's ornaments. He suggested that this process will escalate through positive‐feedback (self‐reinforcement), so that secondary sexual signals become larger and more ‘extravagant’, until the negative trade‐offs on survival exceed the benefits of attracting mates. Fisher's theory of ‘runaway’ sexual selection remained controversial for several years, and even after obtaining theoretical support (Lande, 1981; Kirkpatrick, 1982), as it did not explain why females initially evolve preferences for showy males, or why they do not evolve preferences that would seem more sensible and improve offspring survival.

### (2) Honest indicators of genetic quality

*Females which select males with the most developed characters can be sure that they have selected from among the best genotypes of the male population*. (Zahavi, 1975, p. 207)

Zahavi (1975) dismissed Fisher's runaway sexual selection hypothesis and aimed to provide an alternative explanation. He agreed that conspicuous secondary sexual traits are ‘obviously deleterious to the survival of the individual’ (p. 211), or at least they ‘seem to confer a handicap on survival’ (p. 207). He accepted Darwin's theory of sexual selection (although he later changed his mind (Zahavi & Zahavi, 1997), and he accepted Fisher's claim that conspicuous secondary sexual traits are ‘exaggerated’. However, he objected to the idea that costly traits evolve as a by‐product of runaway sexual selection. Zahavi asserted that we can assume that females are attracted to male sexual displays because these traits allow females to obtain high‐quality mates, and improve the *genetic quality* of their offspring. Zahavi is widely credited for this idea, although it had been suggested previously by Fisher (1930), Williams (1966), and Trivers (1972) (reviewed in Andersson, 1994). Zahavi cited these authors, but not for this idea, which is perhaps why he became credited for indicator or ‘good‐genes’ sexual selection (Section VI). But why should males honestly advertise their quality, and how do females discriminate quality? No one had yet suggested why such signals should be reliable indicators of health and vigour. As Zahavi (1975) pointed out: ‘On one hand, it is a common observation that the most beautiful males of a bird species, or the deer with the largest antlers, are preferred by females, and on the other hand, there is no simple explanation to suggest in what ways the preferred males should be better quality than others’ (p. 205).

Courtship and mating in those days were still widely assumed to be cooperative interactions between males and females, whereas Zahavi emphasized why we should expect deception due to conflict between the sexes. He cited Williams' (1966) description of mate selection as part of the ‘evolutionary battle of the sexes’ and his argument that it is in a male's advantage to ‘pretend to be highly fit whether he is or not’, so that ‘genic selection will foster a skilled salesmanship among the males and an equally well‐developed sales resistance and discrimination among females’ (Williams, 1966, p. 184). As Zahavi stressed, ‘A male may try to cheat a potential female mate so as to increase its chances to get more or better females’ (Zahavi, 1977a, abstract). Zahavi's arguments would help spur interest in sexual conflict, as well as sexual selection, and the problem of explaining reliable communication when there are conflicts of interest between signallers and receivers. This problem with trusting signals when there are conflicts of interest and asymmetries in information was also recognized in economics, where it became known as *cheap talk* (Crawford & Sobel, 1982; Farrell, 1987; Farrell & Rabin, 1996).

### (3) The Handicap Principle: Zahavi's first proposed solution

To explain the evolution of costly secondary sexual traits, and how such traits might provide reliable indicators of quality, Zahavi (1975) suggested that males ‘handicap themselves’ to demonstrate their high quality (p. 212), and that such traits evolve through mate choice because they allow females to ‘select the better male’ (p. 206). ‘Sexual selection is effective’, he argued, ‘because it improves the ability of the selecting sex to detect quality in the selected sex’ (p. 207). Furthermore, he pointed out, ‘Females which choose by a sexually selected character compromise. They select a good quality male which is handicapped but they can be assured as to their mate's quality’ (pp. 207–208). However, rather than viewing the negative fitness effects of a male's sexual display as a by‐product or trade‐off from the interaction between natural and sexual selection, Zahavi argued that sexual selection is ‘effective only by selecting a character that lowers the survival of the individual’ (p. 207).

Zahavi (1975) proposed a simple ‘verbal model’ to explain *how* secondary sexual signals provide reliable signals of quality by reducing survival: he suggested that such signals are reliable indicators of male quality because only high‐quality males are able to survive long enough to breed; low‐quality males that develop the same sexual trait have poor survival, and consequently sexual signals provide a reliable signal of a male's ability to survive. This idea is called the *fixed handicap hypothesis*, but it is also known as *Zahavi*'*s handicap*, the *simple handicap*, the *qualifying handicap*, and the *epistatic handicap*. Zahavi explained his proposal:

*It is possible to consider the handicap as a kind of test imposed on the individual. An individual with a well‐developed sexually selected character is an individual which has survived a test. A female which could discriminate between a male possessing a sexually selected character, from one without it, can discriminate between a male which has passed a test and one which has not been tested. The more developed the character the more severe was the test. Females which selected males with the most developed characters can be sure that they have selected from among the best genotypes of the male population*. (Zahavi, 1975, p. 207)

Zahavi considered the kinds of information that males might signal to potential mates, and he argued that it should not be arbitrary, and such traits should assist females in assessing aspects of quality that have particular ecological importance for their species:

*The handicap principle as understood here suggests that the marker of quality should evolve to handicap the selected sex in a character which is important to the selecting sex, since the selecting sex tests, through the handicap, the quality of its potential mate in characters which are of importance…The adaptive significance of the attracting character should lower the fitness of the selected sex in relation to the main ecological problems of the species. The selecting sex should be attracted by a marker only when the handicap it imposes on its mate and its offspring is smaller than the advantage gained by securing a better (tested) mate*. (Zahavi, 1975, p. 213)

If a male's secondary sexual display functions, for example, to ‘show off his prowess’ and ‘his ability to waste energy,’ (p. 213) then energetic efficiency should be of particular ecological importance to females of this species. Zahavi argued that animals should evolve ‘*particular* patterns to signal *particular* messages’ (Zahavi, 1978, p. 182).

Zahavi (1975) suggested that his Handicap Principle explains the evolution of several puzzling and seemingly costly or wasteful traits. For example, he argued that the ‘excessive tail plumes of the peacocks which seem to attract the females are obviously deleterious to the survival of the individual’ (p. 211). He proposed that only high‐quality males are able to survive the burden of these handicaps, and that females that select males with long plumes therefore obtain high‐quality males. He recognized that ‘it would certainly be better for females to choose high‐quality males which were not handicapped by the plumes’ (p. 211). He argued that we can assume that the function of the plumage of peacocks is to make it easier for females to discriminate male quality. Similarly, he suggested that males in lekking species perform elaborate courtship displays because such behaviours are risky and attract predators as a way to show females the amount of risk that they can afford to take and still survive. He proposed that a male's fighting ability and territory size also provide a reliable ‘index’ of their quality (p. 212). ‘If displays had evolved to communicate in the most efficient way the whereabouts of a bird, in saving energy and reducing predation hazard, as many alarm calls have evolved, then they would not serve as markers of quality’ (p. 211). Rather than using deceptive tricks to hide defects, Zahavi argued that males evolve signals that draw attention to their inadequacies, as a way to show that they are honest about their quality (Zahavi, 1978, p. 183).

### (4) Criticisms of the handicap hypothesis

Zahavi's (1975) paper initially received many criticisms, and critics complained that his arguments were unclear, unconvincing, and logically flawed. Maynard Smith (1976) emphasized that it is not obvious whether the advantages that choosy females gain by conferring traits that improve offspring quality will outweigh the disadvantages from their inheriting ‘handicaps’ that reduce survival. This problem is a special case of a more general problem of explaining the evolution of traits under selection that have conflicting effects on fitness (i.e. antagonistic pleiotropy). Maynard Smith's criticism provided a major challenge to the handicap hypothesis, and he recognized that verbal arguments are inadequate to resolve this matter.

Several theoretical models attempted to test Zahavi's verbal model (fixed handicap hypothesis), but none provided support. (*i*) The first model confirmed that choosy females will incur a fitness disadvantage by producing offspring carrying costly secondary sexual traits, and that any advantages that choosy females potentially gain soon disappear, so that such mating preferences become a disadvantage (Davis & O'Donald, 1976); (*ii*) A second model considered the evolution of sex‐limited signals, so that daughters of choosy females inherit their father's high quality without his costly ornaments, but this version did not work either (Maynard Smith, 1976); and (*iii*) A third model confirmed that this model does not work, although suggested that it modulates the dynamics of runaway sexual selection (Bell, 1978). These models supported Fisher's idea that once females evolve a preference for a male trait, then these traits undergo reinforcing sexual selection, and it was suggested that simulations need to control such ‘Fisher effects’ to test other explanations for sexual selection (Maynard Smith, 1976; Bell, 1978). It was generally concluded that the logic of Zahavi's handicap hypothesis is flawed.

Dawkins (1976) pointed out why Zahavi's arguments are flawed: they assume that costly signals evolve *because* rather than in spite of their costs. The logical conclusion is that selection should favour ‘the evolution of males with only one leg and only one eye’ (p. 172). Costly secondary sexual traits can be favoured by selection but only as long as their reproductive benefits exceed their viability costs, as Darwin (1874) pointed out. There can be no selection *for* a handicap, contrary to Zahavi's claim. Moreover, Zahavi's arguments are circular: he began arguing that costly signals evolve because they are reliable indicators of quality, and then he concluded that reliable signals evolve because they are costly. The circularity of Zahavi's argument has not been pointed out previously, at least to our knowledge, although this is what makes his arguments so confusing and Necker‐cubish.

In summary, Zahavi (1975) aimed to explain the evolution of conspicuous secondary sexual characters, which he assumed provide costly and reliable indicators of genetic quality that evolve through sexual selection. He proposed that costly secondary sexual signals evolve as a mechanism to demonstrate their reliability (general handicap hypothesis), and he proposed a verbal model to explain how his hypothesis might work (fixed handicap hypothesis). His arguments turn Darwinian logic upside down, and theoreticians showed that his verbal (fixed) handicap model does not work. Zahavi remained undaunted, however, and he continued to try to convince the scientific community about the logic of his Handicap Principle.

## IV. ZAHAVI'S CLARIFICATIONS OF HIS HANDICAP PRINCIPLE

*The handicap principle is a very simple idea: waste can make sense, because by wasting one provides conclusively that one has enough assets to waste and more. The investment – the waste itself – is just what makes the advertisement reliable*. (Zahavi & Zahavi, 1997, p. 229)

To address his critics, Zahavi attempted to clarify his claims for his handicap hypothesis (Zahavi, 1977a, 1981, 1987; Zahavi & Zahavi, 1997), and he focused on making the following arguments: (*i*) signals are reliable because they have extra costs that function to show their reliability; (*ii*) signals are honest because they are wasteful, as well as costly; (*iii*) signals evolve under ‘signal selection’, which favours waste rather than efficiency; and (*iv*) the handicap hypothesis provides a general principle to explain the evolution of honest signals (i.e. the *Handicap Principle*). Zahavi's clarifications of his handicap hypothesis have generally been ignored, and therefore, we summarize his arguments and show why they can be rejected.

### (1) Signals have extra viability costs to demonstrate their reliability

Zahavi (1975) argued that sexual signals, such as the tail plumes of peacocks, are ‘excessive’ (p. 211), implying that they are larger and costlier than they need to be to function. He clarified that the handicap hypothesis predicts that males pay ‘extra costs’ for their sexual displays because the ‘extra exertion’ is ‘essential to assure the honesty of the message’ (Zahavi, 1981, p. 135). Such hypothetical costs have been labelled as ‘strategic costs’ and their existence has been proposed to provide the critical prediction for testing the handicap hypothesis (Maynard Smith & Harper, 1995, 2003). The assumption that signals can be costly is testable, however, no one has proposed how to measure any hypothetical extra costs, or how such extra costs might maintain the evolution of signal reliability (Számadó & Penn, 2015). If an individual's investment into a signal *is* the information that is transmitted and assessed by receivers, as Zahavi proposed, then this investment cannot be separated (in theory or in practice) from other ‘minimal costs’ required to produce the signal, contrary to what has been proposed (Dawkins & Guilford, 1991; Maynard Smith & Harper, 1995, 2003).

The handicap hypothesis predicts that secondary sexual traits are reliable indicators of quality because they are costly and reduce survival (Zahavi, 1975). Zahavi also argued that honesty is *enforced* due to increased proximate costs of signal expression (i.e. energetic or other physiological costs, which are more accurately labelled as investments or expenditures), and that such investments necessarily have viability costs (i.e. fitness *trade‐offs*). The vast majority of empirical studies aiming to test the handicap hypothesis have also focused on measuring the proximate costs of signals as a proxy for fitness, which has led to fruitless debates over how high such costs must be in order to support the handicap hypothesis (Kotiaho, 2001; Searcy & Nowicki, 2005). No models exist to offer such predictions, and it is the *net fitness benefits* of signal investment and expression that need to be measured to determine their function. The expression of secondary sexual traits is often positively correlated with individual survival (Jennions, Møller & Petrie, 2001), which is likely because such signals are often condition dependent, i.e. expression depends upon individual quality (see Section V).

### (2) Signals are honest because they are wasteful

Zahavi (1975, 1977a) proposed that signals are reliable because they are wasteful, as well as costly, and that it is their wastefulness that makes them reliable. This argument is a weak version of the handicap hypothesis because it suggests that in order to be reliable, signals only need to be *inefficient*; they do not need to be so costly that they reduce survival. He often argued that energetic or other proximate investments into signalling are sufficient to enforce honesty, and he and others often muddled proximate and ultimate explanations. Nevertheless, Zahavi's argument for waste is central to most of his efforts to explain signal reliability, and he considered it to be the defining feature of his Handicap Principle (Zahavi & Zahavi, 1997).

The notion that sexual signals are excessive, extravagant, and wasteful was not new, but to our knowledge there has never been any evidence for this assumption. Secondary sexual traits had long been viewed as harmful for the long‐term survival of a species, and this assumption motivated part of the resistance to sexual selection (Cronin, 1991). Before the 1980s most still assumed that natural selection maximizes group or species survival, and Darwin's theory of sexual selection seemed maladaptive and wasteful from this perspective. As Lorenz (1963) complained, ‘The evolution of the Argus pheasant has run itself into a blind alley. The males continue to compete in producing the largest wing feathers, and these birds will never reach a sensible solution and ‘decide’ to stop all this nonsense at once’ (p. 37). It would take years for biologists to shake off naive assumptions about group selection, and the belief that sexual selection is a wasteful, maladaptive process because it potentially increases the risk of extinction in the long term.

Zahavi, like some biologists, assumed that natural selection maximizes individual survival, and that secondary sexual traits are wasteful if they impair survival. Characterizing costly signals as ‘wasteful’ or ‘exaggerated’, however, ignores the potential indirect, genetic benefits from investing into signals that can enhance reproductive success (and it ignores the *opportunity costs* of failing to make such investments into signalling). The energy and resources that males invest into the expression of conspicuous sexual signals to attract mates are not necessarily wasteful even if they have negative trade‐offs for survival. Secondary sexual traits seem wasteful if they are harmful to individual survival – but this paradox disappears once we recognize that selection favours lifetime reproductive success (genetic survival) rather than individual bodily survival (Dawkins, 1976). As Cronin (1991) explained, ‘In modern Darwinism, Darwin's contrasts between sexual selection's extravagance, its trade‐offs, its harmfulness, and natural selection's utility, its efficiency, its benefits all melt away. All adaptations are compromises; a trade‐off between mating and predation is no different in principle from a trade‐off between foraging and predation’ (p. 242).

Zahavi's arguments are illogical and contradictory. On the one hand, he argued that signals have extra costs that make them wasteful, but on the other hand he maintained that these additional costs function to demonstrate their honesty. If signals have extra costs that provide an adaptive function, then such ‘costs’ are more accurately described as ‘adaptive investments’ rather than wasteful. His assertion that signals are wasteful is contradicted with his own proposals and it is based on obsolete views of evolution. It assumes that natural selection maximizes individual (or group) survival rather than genetic survival, so that signals or other traits with negative trade‐offs on survival are wasteful – even if they enhance mating and reproductive success. This view of evolution has been replaced in evolutionary biology by life‐history theory and the gene's‐eye view of evolution. Zahavi embraced the assumption that sexual signals are wasteful, and then argued that their wastefulness is functional. He rejected Fisher's runaway sexual selection, and yet he accepted his descriptions of conspicuous male secondary sexual displays as ‘exaggerated’, and that some kind of selection has driven them beyond their optimum for individual survival. Zahavi's handicap hypothesis was inconsistent with Darwinian logic, and he either had to admit that ‘extravagant’ signals are adaptive investments rather than wasteful handicaps, or propose that signals evolve by an alternative mechanism than natural selection. He opted for the latter.

### (3) Signals are wasteful because signal selection favours wastefulness

To explain how selection favours signals because they are costly or wasteful, Zahavi proposed that signals evolve under a special type of selection that he called ‘signal selection’, which unlike Darwinian selection, supposedly favours waste rather than efficiency (Zahavi, 1981, 1987; Zahavi & Zahavi, 1997). Zahavi maintained that ‘According to the theory of signal selection, signals must have a cost in order to be reliable’ (Zahavi, 1987, p. 502). ‘The essence of the theory’, Zahavi (1977b) argued, is that ‘the reliability of communication (or advertisement) is increased in relation to the investment in the advertisement’ (p. 603). Moreover, he argued that animals evolve ‘extra costs’ of signalling to ensure the honesty of the signal (Zahavi, 1981, p. 135). Zahavi (1987) emphasized that ‘the selection of signals is different from the selection of all other characters’ (p. 310). He argued that signals are ‘fundamentally different from the evolution of all other adaptations’ and that they evolve through a special type of selection, which he called ‘signal selection’ (Zahavi & Zahavi, 1997, p. 59). According to Zahavi, signal selection – in contrast to natural selection – favours waste and inefficiency. Furthermore, Zahavi argued that signal selection should replace sexual selection: ‘The sooner that we abandon Darwin's definition of sexual selection and concentrate on understanding the special mechanism of signal selection, the better we shall understand the patterns of signalling systems and the evolution of extravagance’ (Zahavi & Zahavi, 1997, p. 503).

Zahavi never explained how ‘signal selection’ works or how it can favour waste. He merely asserted that it differs from ‘utilitarian’ Darwinian selection because, it ‘results in costly features and traits that look like ‘waste’. It is precisely this costliness, the signaller's investment in the signals that makes it reliable’ (Zahavi & Zahavi, 1997, p. 40). Zahavi recognized that his handicap hypothesis requires a non‐Darwinian explanation, but rather than providing an explanation, he simply coined a new term, ‘signal selection’ (i.e. the nominal fallacy). As one critic pointed out, ‘Costliness is not synonymous with inefficiency’, and ‘it is neither enlightening nor correct to refer to signal selection as selection for inefficiency’ (John, 1997, p. 226). It is difficult to understand why there have not been more criticisms of Zahavi's arguments about wasteful signals and signal selection, especially since they are central to the handicap hypothesis and the entire handicap paradigm.

### (4) The handicap hypothesis is a general principle (Handicap Principle)

Zahavi usually referred to his hypothesis as the ‘handicap principle,’ as he claimed that it provides a general theory to explain the evolution of honest signals. For example, Zahavi (1975) stated: ‘The understanding that a handicap which tests for quality, can evolve as a consequence of its advantage to the individual, may provide an explanation for many puzzling evolutionary problems’ (p. 205). He later clarified that ‘the same principle also applies to male advertising their superiority to intimidate rivals and in fact to all advertisements in general’ (Zahavi, 1987, p. 306). The ‘handicap theory’, he argued, should be ‘applicable to all communication systems’, including inter‐specific communication, and ‘even among cells of multicellular organisms’ (Zahavi, 1977a, p. 258). He proposed that this principle applies to human communication and explains the evolution of altruism (Zahavi, 1977a), language, and art (Zahavi, 1978). Zahavi maintained that he had discovered a unifying scientific principle for explaining biological communication (Zahavi & Zahavi, 1997, p. XVI).

**Figure 2:**
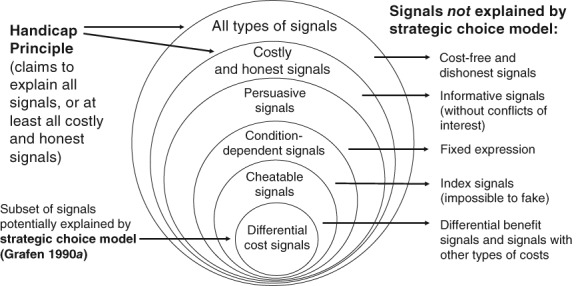
Limitations of Grafen's (1990a *)* strategic choice model for explaining honest signals. Zahavi (1975) originally proposed the Handicap Principle to explain signals that are costly and honest, but then argued that it explains all types of signalling systems, even communication between cells of multicellular organisms (Zahavi, 1977a). Grafen recognized that his strategic choice model only applies to signals that involve conflicts of interest, but he assumed that it explains all such ‘persuasive signals’. However, his model is also limited to signals with condition‐dependent (phenotypically plastic) expression and with differential, marginal fitness costs. It has been suggested that it does not apply to signals that are impossible to cheat (index signals) (Maynard Smith & Harper, 1995), secondary sexual signals (Nöldeke & Samuelson, 2003), and other signals with multiplicative fitness costs and benefits (Getty, 2006). Honest signals can be explained by differential fitness benefits (Godfray, 1991; Johnstone, 1997) rather than costs, and there are other stable signalling equilibria (Lachmann *et al*., 2001; Zollman *et al*., 2013). Critics have emphasized these and other limitations of Grafen's model, but overlooked its differences from the Handicap Principle. In particular, Grafen's model provides an adaptive hypothesis for honest signalling, and it does not assume that honest signals are wasteful or that they evolve because they reduce survival.

The claim that the handicap hypothesis provides a general biological principle for honesty in all types of signalling systems can be rejected for several reasons: (*i*) the handicap hypothesis is limited to explaining signal honesty under conflicts of interest. To address this criticism, Zahavi maintained that conflicts of interest are intrinsic to ‘all communication systems’ (Zahavi, 1977a, p. 258), but this claim is easily rejected; (*ii*) Contrary to its central prediction, theoretical analyses have shown that signal costs *paid at equilibrium* are neither necessary nor sufficient to explain the evolution of signal reliability (Hurd, 1995; Getty, 1998a; Számadó, 1999; Lachmann, Számadó & Bergstrom, 2001); (*iii*) There are many anomalies and alternative explanations for signal reliability (Maynard Smith & Harper, 1995, 2003); (*iv*) Despite decades of research, unequivocal empirical evidence is lacking for the handicap hypothesis (Searcy & Nowicki, 2005); and (*v*) Support for the handicap hypothesis is based entirely on theoretical models, and especially Grafen's (1990a) strategic choice model, which is too restricted to provide a general principle (Getty, 1998a,*b*; Számadó, 2000) (see Section VII.6 and Fig. 2), and moreover, it is not a handicap model, as we show below (Section VII.4 and 5). Thus, we can confidently rule out Zahavi's Handicap Principle.

### (5) Veblen's canon of conspicuous consumption

*In order to gain and to hold the esteem of men it is not sufficient merely to possess wealth or power. The wealth or power must be put in evidence, for esteem is awarded only on evidence*. (Veblen, 1899, p. 36)

Zahavi's Handicap Principle was not completely novel, as a very similar idea had been proposed previously in the social sciences. Thorstein Veblen (1899) was baffled at the ostentatious displays of wealth by the *nouveau riche*, and their tastes for expensive clothing, jewellery, and art, because such expenditures seem to have no utilitarian benefits. As an economist, he was puzzled why rich people waste their time on leisurely pursuits, and squander their money on luxury goods that are neither useful nor productive. In his book, *The Theory of the Leisure Class*, Veblen proposed that the upper class signal their status through the visible or public displays of goods and services, which he called ‘conspicuous consumption’. Such displays provide reliable status signals, he argued, because they are costly and only the wealthy can afford to squander time and money. Women hamper themselves with long fingernails, he proposed, to demonstrate that they are ‘kept’ wives of the leisure class. Veblen argued that in order to be reputable, consumption had to be conspicuous and wasteful, and he called his hypothesis, the *canon of conspicuous consumption*. Veblen's arguments for explaining costly and reliable signals of status are virtually identical to Zahavi's handicap hypothesis (and his writing is also more like a work of art than science and open to many interpretations).

Veblen's arguments that status signals are wasteful, over‐investments were also based on implicit assumptions about the level of signalling that is optimal for individuals or the species (human welfare), and he did not try to conceal his contempt for conspicuous consumption. High levels of consumption may be harmful for humanity, but this does not mean that such behaviour is necessarily wasteful or maladaptive for individuals. If conspicuous consumption allows individuals to maintain their status, then such behaviour may be functional, regardless of its impact on humanity or the planet. To his credit, Veblen conceded that he used the term *waste* only ‘for want of a better term’, and that it is potentially a misleading term because, while such expenditures do not serve humanity, they might have benefits (utility) for individuals (p. 97–98). He continued to use the term ‘wasteful’, however, and he did not explain what maintains signal reliability – if not wastefulness. He did not explain why the wealthy do not purchase *useful* items that the lower classes would also not be able to afford, instead of luxury goods. Thus, Veblen did not explain why people acquire and display luxury goods.

Nevertheless, Veblen's ideas have been valuable for spurring interest in explaining the consumption of luxury goods (now known in economics as *Veblen effects*, *snob appeal*, and *bandwagons*) (Leibenstein, 1950; Bagwell & Bernheim, 1996). Why are people willing to spend more money on items because rather than despite that they are expensive or popular? Economists' efforts to explain behaviours that seem irrational and wasteful for individuals is similar, if not identical, to the challenge of explaining conspicuous secondary sexual displays. Fashion and runaway consumption in human societies have much in common with sexual selection. Consumers that purchase best‐sellers, top‐20 hits, and fashionable clothing merely because they are popular behave much like peahens, whose preferences evolved through runaway sexual selection (Dawkins, 1986). Veblen attempted to integrate economics with evolutionary analyses, but he did not consider sexual selection. There is increasing interest in the consequences of runaway consumerism for humanity, and manipulation by advertising (Durning, 1992; Frank, 2000; Akerlof & Shiller, 2015). Unfortunately, sexual selection theory and other insights from evolutionary biology into over‐consumption have been generally ignored (Penn, 2003). Incidentally, Lorenz's (1963) comments about the wastefulness of sexual selection cited above (Section IV.2), were not criticisms of sexual selection theory, contrary to what has been suggested (Cronin, 1991). Instead he warned about the harmful consequences of sexual selection for humanity, including aggression, runaway consumerism and other wasteful competition (e.g. Lorenz cited Vance Packard's book *The Hidden Persuaders* to show how consumers are vulnerable to deception and manipulation from the advertising industry).

Veblen's ideas surely paved the way for the Handicap Principle. It is often assumed that Zahavi developed his ideas independently from Veblen, as Zahavi did not cite him, at least for many years, and even then he did not mention the similarities between their arguments (Zahavi & Zahavi, 1997). However, Veblen's conspicuous consumption was well known among academics, and especially among critics of consumerism (Zahavi was a conservation biologist, and one of the founders of the Society for the Protection of Nature in Israel). Regardless, Veblen's conspicuous consumption does not provide support for Zahavi's Handicap Principle, nor *vice versa*, as their arguments are flawed for many of the same reasons.

### (6) Summary: the fall and resurrection of the Handicap Principle

In summary, Zahavi (1977a, 1981, 1987) attempted to clarify and explain his handicap hypothesis and why it provides a general principle for reliable signalling. He argued that signals are wasteful, as well as costly, which ensures their reliability and makes them beneficial. He realized that his theory requires a non‐Darwinian type of explanation; but his ‘signal selection’ was just a different name for the Handicap Principle. Zahavi's arguments are circular, contradictory, and incompatible with Darwinian logic. And yet, without any empirical evidence whatsoever, the Handicap Principle soon became transformed from being dismissed as ‘laughable nonsense’ to becoming ‘the central explanation underlying all forms of animal communication’ (Pomiankowski & Iwasa, 1998, p. 928). This transformation was due to Grafen's (1990a) persuasive paper, which reportedly vindicated the Handicap Principle (Fig. 1). However, his model does not vindicate the Handicap Principle, although as we will show, it does support another hypothesis that Zahavi (1977b) proposed. Next, we examine this hypothesis and we show how it differs from the Handicap Principle.

## V. ZAHAVI'S ADAPTIVE CONDITION‐DEPENDENT SIGNALLING HYPOTHESIS

*The phenotypic manifestation of the handicap is adjusted to correlate to phenotypic quality of the individual … Further, it is reasonable to assume that high‐quality phenotypes and experienced individuals pay less for the cost of the same sized handicap than low‐quality phenotypes*. (Zahavi, 1977b, p. 603)

### (1) Zahavi's second hypothesis for honest signals

While attempting to defend and clarify his Handicap Principle, Zahavi (1977b) proposed another explanation for the evolution of reliable signals. In a two‐page letter to the *Journal of Theoretical Biology*, Zahavi argued that his critics had relied on ‘simple mathematical models’ to test his (fixed) handicap hypothesis, and that they had overlooked more sophisticated types of signal expression (p. 603). He suggested that males adjust the expression of their sexual displays according to their quality (condition‐dependent signalling), and that they do this because low‐quality males pay higher fitness costs for signalling than high‐quality males (i.e. differential, marginal costs). This way, he reasoned that ‘the handicap as a marker of honest advertisement (communication) may have its adaptive value with very small cost’ (p. 604). ‘The essence of the theory’, Zahavi proposed, is that ‘the reliability of communication (or advertisement) is increased in relation to the investment in the advertisement’ (p. 603). Zahavi (1981) later clarified that ‘Signals will be reliable only when the signaller of a false message would have to invest more in signalling than it could gain from using a false signal. A reliable signal is thus a signal which is involved with a differential cost, being more costly to a cheater than to an honest individual’ (p. 134). Zahavi did not suggest a name for his new hypothesis, nor did he explain how it might be related to his Handicap Principle; he merely mentioned that it may ‘allow for the widespread use of handicaps in nature’ (p. 603).

Zahavi's second hypothesis for honest signals was not completely novel, as it had been suggested previously that males' secondary sexual traits are expressed in proportion to their phenotypic condition (Fisher, 1915; Williams, 1966; Trivers, 1972) (reviewed in Andersson, 1994). Moreover, Spence (1973, 1974) had previously proposed a nearly identical signalling model in economics (Section VII.2). Nonetheless, Zahavi (1977b) provided compelling arguments for why secondary sexual signals should be condition dependent and reliable indicators of quality, and how condition‐dependent signals can be adaptive. His hypothesis was further developed by Nur & Hasson (1984) and then Grafen (1990a), whose *strategic choice* model is virtually identical to Spence's economic model. Therefore, we refer to Zahavi's second hypothesis as adaptive *condition‐dependent signalling* (Zahavi, 1977b), the *strategic choice* model (Grafen, 1990a), or a *differential cost* signalling model (Spence, 1973) (Section VII.2). *We do not consider this idea to be a type of handicap hypothesis, and we challenge claims that models of this hypothesis provide support for or are equivalent to Zahavi*'*s handicap hypothesis*.

### (2) The many interpretations of Zahavi's second hypothesis

Zahavi's (1977b) second hypothesis initially received a mixed reception: some were optimistic (Eshel, 1978; West‐Eberhard, 1979; Nur & Hasson, 1984; Pomiankowski, 1987), whereas others were sceptical that it could explain the evolution of costly or honest signals (Maynard Smith, 1985; Kirkpatrick, 1986). Many different interpretations, models, and labels were proposed for this idea, including the *quality marker handicap* (Eshel, 1978), the *proportional handicap* (West‐Eberhard, 1979), *conditional handicap* (Maynard Smith, 1985), *courtship handicap* (Motro, 1982), *condition‐dependent handicap* (Pomiankowski, 1987; Grafen, 1990a), and the *strategic choice handicap* (Grafen, 1990a). We reject these labels because Zahavi's second hypothesis is very different from the handicap hypothesis, as we show next. No one to our knowledge has questioned this assumption; it has simply been taken for granted. The only exception is an interpretation called the *revealing handicap* (Maynard Smith, 1985) or *performance handicap* (Hurd & Enquist, 2005), which proposes that condition‐dependent signals are honest because individuals cannot cheat (due to physical, physiological, biochemical, or other proximate constraints). As previously mentioned, it has been suggested that such signals should be reclassified and relabelled as index signals because they are unfakeable and they can therefore be explained without the handicap hypothesis (Maynard Smith & Harper, 1995, 2003). Such traits should be labelled as *putative* indices, however, until a constraint is demonstrated. It has been pointed out that such constraints are potentially adaptive solutions to signalling trade‐offs, however, this does not make them Zahavian handicaps, contrary to what has been proposed (Biernaskie, Grafen & Perry, 2014). Zahavi's (1977b) second hypothesis is incomplete, as it does not explain *how* males adjust the expression of their sexual displays according to their quality. Several additional hypotheses were subsequently proposed to provide proximate mechanisms for adaptive condition‐dependent signalling, such as the so‐called *immunocompetence handicap hypothesis* (Folstad & Karter, 1992) and the *oxidative handicap hypothesis* (Alonso‐Alvarez *et al*., 2007). These proposals confused Zahavi's (1977b) adaptive condition‐dependent signalling hypothesis and index signals, but more importantly, none of these are handicap hypotheses.

### (3) Zahavi's second hypothesis *versus* the handicap hypothesis

Zahavi's (1977b) second hypothesis differs from the Handicap Principle for several reasons. (*i*) It proposes a different proximate mechanism for signal expression (phenotypic plasticity), and a different selective mechanism to explain signal reliability compared to his original (fixed) handicap model (Zahavi, 1975). Rather than proposing that honest signals are a consequence of natural selection eliminating low‐quality signallers from the population, it suggests that reliability is due to condition‐dependent (phenotypically plastic signal regulation), which evolves as an adaptive mechanism to *minimize* the fitness costs or trade‐offs of signalling. Both hypotheses assume differential, marginal fitness trade‐offs for signalling, but otherwise, these hypotheses are very different; (*ii*) His second hypothesis does not assume that signals are wasteful or that costly signals evolve because they are costly or wasteful, and it does not require a special type of selection that favours waste. Instead, it suggests that honest signals are favoured by selection despite their negative effects on survival; (*iii*) It is not the costs of signalling (or wastefulness) that maintains honesty in this scenario, but rather the differential, marginal cost of signalling (Grafen, 1990a; Iwasa, Pomiankowski & Nee, 1991; Hurd, 1995; Számadó, 1999). It is the relative costs of cheating rather than the absolute costs of signalling at equilibrium that enforces honesty (Számadó, 1999, 2000, 2011; Lachmann *et al*., 2001); (*iv*) Zahavi's second hypothesis does not need to provide a general principle to explain honest signals, and it can stand without the handicap hypothesis or the Handicap Principle; and (*v*) His second hypothesis is logical and consistent with evolutionary principles, and moreover, it provides a Darwinian *alternative* to the handicap hypothesis. Thus, it is deeply misleading to equate Zahavi's second signalling hypothesis (or models of this proposal) to the handicap hypothesis (or Handicap Principle).

In summary, Zahavi (1977b) proposed a second hypothesis to explain how honest signals evolve, and it provides a logical and Darwinian alternative to his handicap hypothesis. His second hypothesis received many different interpretations and labels, but they all assumed that this idea is a version or the correct interpretation of the Handicap Principle. It was Zahavi's second signalling hypothesis that Grafen's (1990a) strategic choice model supported; not the handicap hypothesis, as we show below (Section VII). To understand Grafen's interpretations, however, we first examine the early attempts to model Zahavi's (1977b) second hypothesis, which were important for the development of both sexual selection and honest signalling theory.

## VI. SEXUAL SELECTION AND HONEST SIGNALLING MODELS

Finding a model that supports the Handicap Principle became a theoretical challenge in evolutionary biology comparable to constructing a perpetual motion machine (a mythical device that defies the laws of thermodynamics) in theoretical physics. Theoretical analyses of Zahavi's proposals during the 1980s addressed two different, although related issues: (*i*) the evolution of costly and honest secondary sexual signals (sexual selection theory); and (*ii*) the evolution of honest signals in general (i.e. honest signalling theory, also called ‘costly signalling theory’). Researchers used different modelling approaches (population genetics *versus* game theory models), and this research developed largely in parallel.

### (1) Sexual selection models

*[Zahavi*'*s handicap theory] cannot have the effects claimed for it*. (p. 2) *At the theoretical level, present models indicate that, in polygynous species, the process envisaged by Fisher is overwhelmingly more important than any kind of handicap effect*. (Maynard Smith, 1985, p. 4)

Zahavi's proposals generated much interest in sexual selection, and they sparked a major debate between advocates of Fisherian runaway *versus* indicator (or good‐genes) models of sexual selection (Andersson, 1994). Zahavi (1975) failed to provide a convincing explanation for the evolution of male secondary sexual signals, but he attracted much interest in explaining female preferences for such traits – and the idea that female preferences potentially function to enhance offspring genetic quality. Zahavi's (1977b) second hypothesis inspired further theoretical analyses, especially on the evolution of condition‐dependent indicators of genetic quality (Eshel, 1978; Andersson, 1982, 1986; Maynard Smith, 1985; Kirkpatrick, 1986; Pomiankowski, 1987; Michod & Hasson, 1990). Both sides of this debate confused Zahavi's (1977b) second hypothesis with his Handicap Principle, however, and both mistakenly equated the latter with indicator (good‐genes) models of sexual selection – and *vice versa*. The question for sexual selection researchers was not honest signalling *per se*, but rather explaining the selective maintenance of *female preferences* for costly male secondary sexual signals. There was also much interest in the maintenance of heritable variation in male fitness, which is necessary to maintain indirect benefits for female preferences, but it turned out that this issue is not as problematic as once assumed based on overly simple single‐locus models (Rowe & Houle, 1996).

Maynard Smith (1985) provided a review of sexual selection models, but he assumed that all indicator (good‐genes) models are handicap models, and he misinterpreted Zahavi's (1977b) second hypothesis. We assume that when he, like many others, suggested that secondary sexual traits might reflect ‘fitness’, that he was referring to *potential fitness*. He explained how Eshel's (1978) defence of Zahavi's arguments made him realize that he had been too dogmatic in his criticisms of the Handicap Principle. Eshel (1978) had defended Zahavi's (1977b) second hypothesis, but he equated it to the Handicap Principle. Maynard Smith defined ‘handicaps’ as secondary sexual signals that indicate differences in viability, although in the next paragraph he defined this term as traits that reduce survival. He proposed that there are three types of handicap models: (*i*) Zahavi's (1975) *fixed* or epistatic handicap model, which had been previously refuted; (*ii*) the *revealing handicap* model, which Maynard Smith defined as costly signals that all males develop with the degree of expression revealing viability, due to constraints such as physical weakness or poor health; and (*iii*) the *conditional handicap* model, which he defined as costly signals that males develop only if they are high quality (fixed, all‐or‐none expression). His *revealing handicap* assumes that signals are condition dependent due to physical, developmental, or physiological constraints on individuals. This was Maynard Smith's interpretation of the Hamilton & Zuk (1982) hypothesis, despite that these authors had rejected a handicap interpretation of their model. Maynard Smith later regretted his interpretation and, as previously mentioned, he relabelled it as an *index signal* (Maynard Smith & Harper, 1995, 2003). Maynard Smith's *conditional handicap* model assumes dimorphic, constitutive signal expression, which is a useful simplifying assumption for modelling, and this is precisely the assumption that Zahavi (1977b) aimed to address with his second hypothesis. Maynard Smith cited West‐Eberhard (1979) for this model (however, she described Zahavi's second hypothesis more accurately than Maynard Smith), and he confused two issues, i.e. whether honest signals need to be condition dependent to be honest, and whether condition dependence can be an adaptation or a non‐adaptive constraint. Based on his interpretations of these models, Maynard Smith concluded that Zahavi's (1975) handicap hypothesis can be rejected. He had misunderstood Zahavi's (1977b) second hypothesis, and failed to recognize its logic, and yet his interpretations became cited more widely than Zahavi's own description!

The first theoretical analyses to investigate good‐genes models of sexual selection provided mixed results for condition‐dependent signalling, but they assumed that all such models are handicap models. (*i*) Andersson (1982, 1986) conducted simulations of sexual selection in a monogamous mating system (to control for Fisher effects), and found that costly, condition‐dependent signals can evolve when they honestly indicate male genotypic quality. However, his model is not a handicap model, as it assumes that the signal is an honest indicator of quality due to an inescapable constraint (although unlike an *index signal*, signal expression is all‐or‐none); (*ii*) Kirkpatrick (1986) investigated several different genetic models and concluded that ‘the handicap mechanism does not work’. His analyses included the evolution of the condition‐dependent expression of costly signals, as predicted by Zahavi (1977b) and others (West‐Eberhard, 1979; Nur & Hasson, 1984), but the results were not qualitatively affected; (*iii*) Pomiankowski (1987) raised concerns that Kirkpatrick (1986) confounded the effects of the fixed handicap (Zahavi, 1975) and the ‘revealing handicap’ models, and that he had unnecessarily assumed that additive genetic variance in viability quickly vanishes. Pomiankowski assumed that the differences between the Zahavi (1977b) ‘condition*‐*dependent handicap’ and the ‘revealing handicap’ models are unimportant, and therefore, he only analysed the latter. He developed a model that showed that the revealing handicap can work but only under certain conditions, i.e. when the fitness effects of the costs of signalling and viability genes combine non‐multiplicatively (fixed handicap), or the costly signal directly reveals genetic quality (‘revealing handicap’). However, he also found that the costly signalling trait cannot spread if the frequency of the preference in females is below a threshold. He concluded that ‘the handicap principle does work – sometimes’, and that it can cause the runaway exaggeration of male sexual signals and female mating preferences, when the above conditions are fulfilled; (*iv*) Grafen (1990b) published a sexual selection model, which he concluded supports the Handicap Principle, but this was a misinterpretation, as we show below (Section VII); and (*v*) Iwasa *et al*. (1991) found support for Zahavi's (1977b) ‘condition‐dependent handicap’, as well as the ‘revealing handicap’ model, however, their model depends on a dubious assumption about biased mutation pressure affecting viability.

Thus, Pomiankowski (1987) found some support for the ‘revealing handicap’ model, but contrary to what he assumed, this is not equivalent to Zahavi's (1977b) ‘condition‐dependent handicap’ hypothesis, and neither are handicap models. His results were not as influential as Grafen's (1990a) models for several reasons: (*i*) he concluded that his results gave only conditional support for the Handicap Principle (i.e. the costly signal was either lost or went to fixation); (*ii*) He explicitly sided with Fisher and Darwin *versus* Zahavi: ‘In conclusion it would be unwise to assert, as Zahavi (1975) did, that sexual selection through female choice is effective because it allows females to detect a potential mate's heritable quality. Primarily sexual selection is effective, as Darwin and Fisher proposed, because males with more exaggerated secondary sexual characters are more attractive to females, and so mate more frequently’ (p. 140); and (*iii*) He stayed within the bounds of sexual selection and did not advocate the broad generalization of the Handicap Principle as an underlying explanation for explaining signals in nature.

### (2) Honest signalling models

Zahavi's proposals triggered interest in the evolution of honest signals in general, as well as secondary sexual traits, and they inspired the development of so‐called *costly signalling theory*. These models are described more accurately as *honest signalling theory*, however, since most aim to explain honesty and use both costs and benefits. Here, we summarize the first models to investigate the evolution of honest signalling.

#### (a) *Enquist's signalling model: performance versus choice*

Enquist (1985) *established … the foundations of ESS signalling theory* (Grafen & Johnstone, 1993, p. 245).

Enquist (1985) used simple game theoretical models to investigate the problem of signal reliability in the context of aggressive interactions, which is relevant to sexual selection (male–male contests), and also to more general agonistic interactions. He differentiated between variation in signalling due to either variation in ‘performance’ or ‘choice’ of signalling. By ‘performance’ he refers to signals in which reliability is guaranteed, not because of their cost, but because of a causal relationship or constraint between the level of performance of the signal and the information transmitted (i.e. index signals). He cited examples of toads and stags that produce calls that reliably indicate their body size, which are assumed to be due to anatomical constraints (Davies & Halliday, 1978; Clutton‐Brock & Albon, 1979). He emphasizes ‘choice’ as an alternative mechanism, in which different individuals are equally capable of performing the signal, but may differ in motivation, for example. He investigated a model in which weak and strong individuals compete for a resource, but their quality is hidden from observers. There is a pre‐fight communication where opponents can choose between two cost‐free signals. He investigated the conditions in which one of these signals is exclusively associated with the strong type and *vice versa*, thus allowing opponents to assess the type of the signaller correctly from the signal. The stability of this model requires that the social benefits of a weak individual cheating (pretending to be strong, thus obtaining the resource without a fight against other weak individuals) is smaller than the potential cost (fighting strong individuals). This model is a fine example where signalling trade‐offs (differential marginal cost and benefits) can maintain honest signalling with cost‐free signals at the equilibrium. This model of ‘performance‐based signals’ has been re‐interpreted as unfakeable index signals, rather than a handicap model (Maynard Smith & Harper, 2003).

#### (b) *Nur and Hasson's signalling models*

*Our models indicate why in nature, where individual differences exist with respect to ‘condition’ (related to social status, territory, nutritional history), those males most successful at defeating their rivals, or most successful at attracting females, will at the same time be the males demonstrating the highest survivorship* (Nur & Hasson, 1984, p. 295).

Nur & Hasson (1984) helped to clarify Zahavi's second hypothesis, and they proposed how it could be modelled by taking an optimality approach. They used several definitions for the term *handicap*, like Zahavi and most others, and they used them interchangeably, e.g. they defined this term as a signal that requires considerable investment (a proximate definition), a signal with fitness costs (an evolutionary definition), and a signal that is a reliable indicator of condition or quality (good‐genes or indicator hypothesis). They embraced Zahavi's (1977b) second hypothesis and argued that adaptive phenotypic plasticity is the ‘essential ingredient’ that explains signal honesty (p. 276). They also recognized the importance of differential signal costs for maintaining signal reliability, although this point was less explicit. They presented two optimality models to show how selection can favour the evolution of honest and adaptive condition‐dependent signals. In their multiplicative model, the signal has a cost (a decrease in survival) and a benefit for mating success and fecundity. They offered two examples, the size of deer antlers and tail length in birds, and they provided graphic models to show how the evolutionary signalling optimum for male sexual signals would be expected to provide a reliable indicator of their condition. They pointed out that many signals can affect survival or fecundity, but not both, and therefore that this situation requires a different model. In the additive model, they considered an example of the stotting behaviour of gazelles (*Gazella* sp.), which had been proposed to be an honest signal of an individual's condition and its ability to outrun a potential predator. They proposed a model, but conceded that its generality was unclear, requiring obtaining quantitative estimates of the effectiveness of the signal as a deterrent. They also considered the roaring contests of red deer (*Cervus elaphus*) stags as honest signals of fighting ability. They challenged a previous interpretation suggesting that roaring cannot be faked because weak individuals are physically unable to roar (Clutton‐Brock & Albon, 1979), and instead they argued that roaring is not faked because it confers a higher fitness cost for weak than strong individuals. Thus, they confused physical weakness, which is a proximate explanation, with differential fitness cost, which is an evolutionary explanation, and such confusion is still common, as we explain below (see ‘revealing handicap’ or ‘index’, Section VII.3). They concluded that ‘the handicap principle, as we have elaborated it, deserves to be seriously reconsidered by evolutionary biologists’ (p. 295).

Nur & Hasson (1984) helped to clarify Zahavi's (1977b) condition‐dependent signalling hypothesis, but they asserted that it is the correct interpretation of the Handicap Principle, and ignored the differences between these proposals. Their claim is puzzling since they were aware of the problems with Zahavi's handicap logic. They pointed out that Zahavi (1975) argued that many signals evolve and are maintained ‘not despite their being a “handicap” to their bearers, but *because* they are a “handicap”’ (p. 276). They acknowledged that his argument was controversial, but they concluded that the debate was due to misinterpretations of the Handicap Principle by others. Grafen (1990a) later made a similar claim, though Zahavi never agreed to these claims, as we explain below. *Changing the definition of the ‘handicap hypothesis’ to match Zahavi*'*s second hypothesis would not be a problem* per se*, unless it resulted in confusing these very different concepts, which is exactly what happened*.

### (3) Summary

Thus, Zahavi's proposals stimulated interest in sexual selection and honest signals, and while some recognized that his second hypothesis was logical, supporters and critics confused it with the Handicap Principle, which became equated to indicator models of sexual selection (and *vice versa*). Zahavi's (1975) fixed handicap model was shown not to work, and although it was suggested that the revealing handicap might sometimes work, this is not a handicap model. Nur & Hasson (1984) clarified Zahavi's (1977b) second hypothesis and explained why it should work, but they did not test whether it provides an evolutionarily stable strategy (ESS). Enquist (1985) first used game theoretical models to investigate the evolution of honest signals, but he did not test either of Zahavi's proposals. The time was ripe to utilize game theory and sexual selection models to clarify the theoretical issues and put the controversies over the Handicap Principle to rest, but this is not what happened.

## VII. GRAFEN'S STRATEGIC CHOICE SIGNALLING MODEL

*Zahavi*'*s major claims for the handicap principle are thus vindicated* (Grafen, 1990a, Abstract)

Grafen's (1990a,*b*) publications dramatically turned the tide for the Handicap Principle (Fig. 1). He contrasted two different perspectives on animal communication: Zahavi claimed that signals must be honest whereas Dawkins & Krebs (1978) argued that signals should evolve to persuade, deceive, and manipulate rather than provide honest information. Grafen stated that he resolved this conflict by showing that Zahavi's claims for the Handicap Principle are substantially correct. In his first paper, *Biological signals as handicaps*, Grafen (1990a) investigated the evolution of honest secondary sexual signals, and he interpreted the results of his model as providing a general solution for all signalling contexts. His signalling models ignored genetics and did not address whether or how females benefit by mating with high‐quality males or whether their offspring obtain genetic benefits. However, he provided a companion paper that he claimed ‘remedies both defects’ by using population genetics models. He concluded that these models ‘defend the centrality of the handicap principle in sexual selection’ (Grafen, 1990b, pp. 517–518). Grafen's (1990a,*b*) papers are widely credited for having validated the Handicap Principle and for placing this concept on a ‘firm mathematical footing’ (Hammerstein & Hagen, 2005). Here, we critically examine Grafen's interpretations of his model, and the justifications for his conclusions.

### (1) The strategic choice ‘handicap’ model

Grafen (1990a) defined the features that he considered necessary for a trait to be called a ‘signal’, and he used a narrow definition that included only traits that provide information about an individual that is hidden and that receivers cannot perceive directly. Traits that advertise an individual's quality are unnecessary if their quality can be perceived directly, which make such traits less interesting, as Spence (1973) argued. Grafen also distinguished ‘persuasive signals’ that evolve under conflicts of interest *versus* ‘informative signals’ that do not, as the challenge is explaining honest signals when there are conflicts of interest that make deception beneficial. In his strategic choice model, he assumed that low‐quality males pay a higher cost for the same investment than high‐quality males (differential cost assumption), and that males (somehow) know their own quality and are able to allocate investment into sexual signals depending upon their quality (adaptive or ‘strategic’ investment). Grafen derived three ‘main handicap’ results: (*i*) signals are honest; (*ii*) signals are costly; and (*iii*) signals are costlier for low‐quality than high‐quality individuals. The third assumption is the critical ‘differential cost’ criterion: ‘Each male's level of advertising is evolutionarily stable, yet the better male advertises more. It follows that the marginal cost of advertising must be lower for the better males’ (p. 521). Grafen concluded that his game theoretical models vindicate Zahavi's Handicap Principle:

*Persuasive signalling necessarily involves waste, as only costs enforce honesty. Further, the costs must be differential, so that it costs a better male less to make the same signal. Although the purpose of the signalling is persuasion from the signaller*'*s point of view, the evolutionary end result is that signalling is honest and the receiver forms a correct opinion of the signaller*'*s quality. These conclusions may be attributed to* Zahavi (1975, 1977; 1987)*. The evolutionary stability of persuasive signalling necessitates honesty, which necessitates waste*. (p. 532).

*If we see a character which does signal quality, then it must be a handicap. The handicap principle lies at the heart of evolutionary signalling, and must therefore play a major role in our understanding of it*. (p. 521).

*The cost of the signal is therefore essential to its operation. It therefore makes sense to say that the reason males signal in this way is because it is costly. The signal is selected because it reduces the fitness of its bearer*. (p. 520).

*The biologically important conclusions from these signalling models are those drawn by* Zahavi (1975, 1977, 1987). (p. 541).

### (2) Similarities to Spence's signalling model

Grafen compared his model to previous signalling models in economics, including a model by Spence (1973, 1974), but he under‐estimated their similarities. Spence considered the practical problem that employers usually face when seeking to hire employees due to having incomplete information about an applicant's qualifications (i.e. their quality). He recognized that some attributes, such as height and sex, cannot be faked by applicants (he called such signals *indices*, but he considered them to be uninteresting because they cannot be faked), and he wondered how employers can otherwise judge an applicant's abilities when they have incomplete information. His model assumes that applicants vary in their abilities to do the job (they are either low or high quality) and that employers pay higher salaries to high‐quality individuals. It also assumed that low‐quality applicants pay higher opportunity costs, which include monetary, time, and effort, for obtaining a higher education than do high‐quality individuals (i.e. they have differential marginal costs or trade‐offs for investing into the signal). As Spence stated, this assumption is critical: ‘It is not difficult to see that a signal will not effectively distinguish one applicant from another, unless the costs of signalling are negatively correlated with productive capability’ (Spence, 1973, p. 358). His model is known as a ‘job‐market’ or ‘job‐screening’ signalling model. It shows that education level can be a reliable indicator of an applicant's abilities, even though it does not improve their actual abilities or productivity, because high‐quality applicants are better able to invest more into education than low‐quality individuals. Riley (1979) extended Spence's findings, and he emphasized the critical importance of differential marginal signalling costs for education level to provide a reliable signal of quality (p. 335), as he explained: ‘Assumption 5 is crucial. The opportunity cost, in monetary units, of increasing the level of the signal *y* is (–U2/ U3). Without the restriction that this be smaller for those capable of producing a higher quality product (those with higher θ), it would always pay those with lower θ to mimic, and thereby obtain the higher price’. Spence's signalling model was well known in economics (in 2001, he shared a Nobel Prize in economics for his analyses of markets with asymmetric information), although his paper had not yet been cited in any biological journals. Grafen introduced these models to biologists, however, he described them as ‘fundamentally different’, and he asserted that ‘despite formal similarities, the biological models and Riley's model provide little mutual enlightenment’ (p. 537).

### (3) Comparisons with other putative handicap models

Grafen also compared his model to previous interpretations of the Handicap Principle (i.e. three putative handicap models proposed by Maynard Smith (1985). First, he compared his model to Zahavi's (fixed) handicap model, which had previously been rejected (Section III), and he emphasized their differences. They both assume differential signalling costs, but otherwise they are very different (Section V.3). Second, Grafen considered the so‐called revealing handicap model, and again he emphasized their differences. He interpreted this model as predicting that males honestly demonstrate their quality by undertaking ‘some onerous task’, and he argued that this model does not operate as a signal because ‘the content of the message is directly observed’ (p. 538). His interpretation differs from Maynard Smith (1985), who proposed this model to explain *signals* that are honest because individuals are unable to cheat due to physical or other constraints (i.e. index signals). Just how Grafen's model differs from index signals is controversial. It has since been suggested that these two hypotheses cannot be discriminated theoretically or empirically (Collins, 1993), and that they are not alternatives but rather two ends of a continuum (Holman, 2012; Biernaskie *et al*., 2014; Biernaskie, Perry & Grafen, 2018). Third, Grafen compared his model with Zahavi's (1977b) ‘condition‐dependent handicap,’ but he underestimated their similarities. He assumed that Zahavi's second hypothesis does not allow flexible signalling by low‐quality males, so that ‘only high‐quality males are capable of expressing the handicap’ (p. 539). His interpretation closely matches Maynard Smith's (1985) conditional handicap model [which was an over‐simplification of West‐Eberhard's (1979) description of Zahavi (1977b)]. However, Zahavi (1977b) had clearly proposed that males adaptively adjust the expression of their sexual signals depending upon their condition, and that the ‘phenotypic manifestation of the handicap is adjusted to correlate with the phenotypic quality of the individual’ (pp. 603–604). Thus, we see no distinction between Grafen's strategic choice handicap model and Zahavi's second hypothesis, which is why we refer to them collectively as *condition‐dependent* or *strategic choice* signalling (Zahavi, 1977b; Grafen, 1990a).

Grafen (1990a) then concluded that his model supports Zahavi's Handicap Principle, and moreover, he argued that ‘This shows that the models given in this paper really are models of Zahavi's handicap principle’ (p. 541). *This was a major leap in logic*. Rather than acknowledging the profound differences between these ideas (Sections V.3), Grafen asserted that these concepts are equivalent, and that ‘there are signs in Zahavi's papers that the strategic choice handicap is indeed what he mainly had in mind’ (p. 539). It is impossible to know what Zahavi mainly had in mind, but he never agreed to this suggestion, and he never placed any particular emphasis on his second hypothesis or Grafen's model. In their book, *The Handicap Principle*, the Zahavis only mention Grafen's model in one sentence and merely to explain how this idea finally became accepted: ‘…Alan Grafen published two papers using different mathematical models to show that the Handicap Principle is generally applicable, and that it is a sound principle that can ensure reliability in communication between competing organisms’ (Zahavi & Zahavi, 1997, p. XV). There are similarities between Grafen's model and the Handicap Principle, but their differences are far more important than their commonalities (Section V.3).

### (4) Re‐evaluating Grafen's justifications for handicap interpretations

Grafen (1990a) provided three arguments to justify why his findings vindicated the Handicap Principle: First, he argued that his model shows that signals are honest because they are costly to produce, and that signal costs enforce honesty (p. 532). Moreover, he asserted that: ‘If advertising were not costly, then the signal could not operate in this way; nor if it were equally costly to good and bad males’ (p. 520). However, he provided no evidence for this interpretation, and subsequent analyses have shown that equilibrium costs of signalling are neither sufficient nor necessary to enforce honesty (Hurd, 1995; Számadó, 1999; Lachmann *et al*., 2001). Honesty in Grafen's model is due to a particular assumption, i.e. marginal and differential trade‐offs between high‐ and low‐quality males. Honesty is maintained by the fitness costs of cheating rather than signalling (Számadó, 2000). Reliable signals can also evolve in a differential benefit model (Godfray, 1991; Getty, 1998a; Godfray & Johnstone, 2000) and neither equilibrium signal costs nor differential costs are required for the model to work this way (see Section VII.6). Second, Grafen concluded that ‘the evolutionary stability of persuasive signalling necessitates honesty, which necessitates waste’ (p. 532). He also asserted that ‘All males voluntarily pay higher advertizing costs than they need’ (p. 520). He suggested that males are free to invest into signals at any level; however, he did not state what level they *need*. He argued that males evolve signals that are wasteful, and that they do not need to attract females, but this is incorrect. Grafen concluded that his model ‘affirms Zahavi's (1987) claim that natural selection on a wide class of signals necessarily incurs waste in accordance with the handicap principle’ (p. 518). However, if signal costs are the investment needed to attract females, as assumed in his model, then it is misleading to describe these costs as ‘unnecessary’ or as ‘wasteful.’ Third, Grafen concluded that costly signals evolve, not despite their costs, but *because* they are costly and reduce fitness. For example, he stated: ‘The signal is selected because it reduces the fitness of its bearer’ (p. 520). This claim is incorrect, and in fact, Grafen immediately qualified his statement: ‘More precisely, it reduces one component of the bearer's fitness and the over‐compensating increase in the other component depends upon the interpretation by females of the signal’ (p. 520–521). This third justification was incorrect, and yet it was the crucial criterion that inspired Dawkins to conclude that Grafen's model was ‘full‐bloodied Zahavian’ (Dawkins, 1989) (p. 312). Thus, none of the justifications that Grafen provided for equating his model to the handicap hypothesis stand up to scrutiny.

### (5) The validity of Grafen's main handicap results

The equations for Grafen's (1990a) strategic signalling model do not stand up to scrutiny either, as they do not support his main handicap conclusions. Conclusions were made about the *absolute costs* of signalling at the equilibrium, as predicted by Zahavi's handicap hypothesis, but the equations only provide predictions about the *marginal costs* of signalling. In other words, the second and third conclusions of the main handicap results are incorrect according the equations for the model. Moreover, subsequent investigations have confirmed that these conditions are not the general conditions of honest signalling: (*i*) Signals need not be costly at the equilibrium to be honest not even under conflict of interest nor under the assumption of costly signalling (Hurd, 1995; Számadó, 1999; Lachmann *et al*., 2001; Számadó *et al*., 2019); and (*ii*) Signals need not be costlier for worse signallers (Getty, 1998a, 2006). Thus, Grafen's model does not offer any predictions about signalling costs at the equilibrium. The reason that Grafen predicted a positive equilibrium cost is due to an external assumption, namely that the equilibrium cost of signals for the worse quality signallers is zero. This may be a biologically realistic assumption, but it is not a necessary aspect of the logic of honest signalling. With this assumption, Grafen excluded all the possible solutions in which the costs of equilibrium signals are zero or negative. Thus, he concluded that costly signalling is an integral part of the logic of honest signalling, as if it follows from his equations, but it is not. This model does not provide a general principle for honest signalling.

### (6) Additional limitations of the strategic choice signalling model

There are additional reasons why Grafen's (1990a) signalling model lacks generality (Fig. 2). First, differential costs are not necessary to enforce signal reliability, as signal reliability can be explained by differential *benefits* (i.e. high‐quality individuals obtaining greater fitness benefits than low‐quality signallers) (Godfray, 1991; Getty, 1998a; Godfray & Johnstone, 2000). Differential benefit models have been labelled as ‘general handicaps’ and they have been interpreted as generalizing Grafen's strategic choice model to other signalling contexts (Johnstone, 1997; Laidre & Johnstone, 2013). It is misleading, however, to refer to differential benefit models as ‘handicap models’ or ‘costly signalling theory.’ We propose that differential cost and benefit models are ends of a continuum, so that the selective advantage of a signal, as for any other trait, should be due to the net fitness benefits (and cost/benefit trade‐offs). Second, it has been suggested that Grafen's model is limited to signals with additive costs and benefits, which would not be expected to apply to secondary sexual signals having multiplicative trade‐offs (i.e. the costs of signalling affect survival whereas the benefits influence reproduction) (Getty, 1998a, 2006). This criticism deserves additional attention in the future. Third, economists have found that such signalling models have a multiplicity of equilibrium outcomes, and that they do not provide a general explanation for reliability (Fudenberg & Tirole, 1991). Finally, Grafen's (1990a) signalling model does not address whether females obtain genetic benefits by mating with high‐quality males, and his companion paper does not remedy this defect, as we show next.

### (7) Grafen's population genetic model of sexual selection

*The main biological conclusions of this paper are the same as those of Zahavi*'*s original papers on the handicap principle* (Grafen, 1990b, p. 487).

In Grafen's (1990b) companion paper, *Sexual selection unhandicapped by the Fisher process*, he provided a population genetic model in which he investigated sexual selection while controlling for Fisher effects. He described them as ‘genetic models of signalling.’ The males differ in their quality and signal their quality through sexual advertisements. Females assess the males' signals, and prefer to mate with high‐quality males. He concluded that these models ‘demonstrate the logical coherence of Zahavi's (1975, 1977, 1987) Handicap Principle in the context of sexual selection’. Moreover, he argued that these models place Zahavi's Handicap Principle on the ‘same logical footing as the Fisher process, in that each can support sexual selection without the presence of the other’ (Grafen, 1990b, p. 473).

In these models, females can only evaluate male quality by their advertisement, and male advertising level is either unconditional or conditional, so that there can be an increasing or decreasing function of quality. Advertising reduces male survival, and especially for low‐quality males (differential viability costs), and male quality is *environmentally determined* to make the model analytically tractable. Females can increase their *fecundity* by mating with males of higher quality. Choosy females thus obtain direct fitness benefits, but there are no indirect, genetic benefits or costs. The model does not address whether the benefits of mating with high‐quality males outweigh the disadvantage incurred by sons carrying detrimental secondary sexual traits, which was Maynard Smith's (1976) original objection to the Handicap Principle. Grafen (1990a) acknowledged the necessity of examining net viability for his signalling model, and he stated that when females pay the ‘full costs of the handicap’, it lowers the level of advertising, although otherwise the results are unchanged (p. 525–526). This model could not examine the ‘full costs’ to choosy females, however, as it is not a genetic model. In his companion sexual selection paper, Grafen (1990b) stated that he and Greenough were working on a sexual selection model in which quality is genetically determined (p. 475), but to our knowledge, this model is still unpublished.

The net viability issue is a major gap in Grafen's sexual selection model, and also in subsequent models of strategic choice signalling. Only one study has addressed this issue to our knowledge: Nöldeke & Samuelson (2003) pointed out that Grafen (1990a) circumvented this issue by ‘working with a ‘model of the [strategic] model’ that implicitly assumes the existence of an equilibrium in the underlying strategic model (Grafen, 1990b, pp. 515–516)’ (p. 58). They constructed a game‐theoretic strategic choice handicap model, in which males produce costly signals to advertise their quality to females. Females benefit from net viability of the males, which is a function of the male quality and their signals. They found a signalling equilibrium, but only if females in their model do *not* bear the cost of male advertising. Their results show that when females' fitness depends on males' net viability, the existence of a signalling equilibrium cannot be taken for granted. Nevertheless, they acknowledged that this model might still be useful for investigating other signalling contexts, such as predator–prey interactions (e.g. see Yachi, 1995).

### (8) Summary: towards unhandicapping honest signalling and sexual selection theory

In summary, Grafen (1990a,*b*) investigated models on the evolution of honest signals of quality, but these models have been misinterpreted for several reasons. (*i*) The strategic choice signalling model was not as novel as often assumed because it is a model of Zahavi's (1977b) second signalling hypothesis and it is nearly identical to a previously published model in economics (Spence, 1973, 1974; Riley, 1979); (*ii*) Grafen's models do not vindicate the Handicap Principle, contrary to what has been widely assumed. These conclusions were based on Zahavi's mistaken claim about the necessity of equilibrium signal costs for honest signalling, and misinterpretations that Grafen's model validates this claim. Grafen's main results provide no general predictions about the necessity of equilibrium signal cost as a condition of honest signalling. Thus, Grafen's ‘main handicap results’ are not the correct interpretation of these equations; (*iii*) A biologically realistic but external assumption was introduced to obtain costly equilibria, yet Grafen incorrectly attributed such costliness to his equations (i.e. ‘main handicap results’); (*iv*) The possibility that there are other evolutionarily stable equilibria, cost‐free or partially honest (Lachmann *et al*., 2001; Zollman, Bergström & Huttegger, 2013) was not investigated, and therefore it cannot be concluded that the stable evolutionary equilibrium for all signals is costly and honest; (*v*) Grafen's (1990b) population genetic model did not address whether the benefits of mating with high‐quality males outweigh the disadvantages incurred by sons carrying detrimental secondary sexual traits (Nöldeke & Samuelson, 2003); and (*vi*) Grafen's models were over‐generalized to apply to all types of honest signalling with conflicts of interest (Fig. 2).

Rejecting the Handicap Principle has many important implications for sexual selection, as well as honest signalling theory, and we propose that researchers avoid labelling secondary sexual signals as ‘handicaps’ and good‐genes models of sexual selection as ‘Zahavian’. Zahavi had a very important influence on the development of sexual selection theory, but he was not the first to propose that secondary sexual traits are honest indicators of genetic quality. Moreover, he proposed his Handicap Principle as a mutually exclusive alternative to Fisherian sexual selection, and he later argued that sexual selection should be abandoned and replaced by signal selection (Zahavi & Zahavi, 1997). It has been suggested that the debate over Fisherian *versus* good‐genes models for explaining mating preferences is a false dichotomy (Eshel, Volovik & Sansone, 2000; Kokko, 2001), and that a unification of these models is needed and should be labelled the ‘Fisher–Zahavi’ model in honour of Zahavi's contributions (Kokko, 2001; Kokko *et al*., 2002, 2003). We agree that Fisherian and good‐genes models are not mutually exclusive, but nevertheless it is useful to distinguish between traits that affect reproductive success *versus* those that influence viability. Indeed, trade‐offs between these two traits are central to evolutionary life‐history theory, and Grafen's (1990a) strategic signalling model is better understood as a life‐history model in which individuals differ in quality and optimally allocate resources into traits for survival *versus* sexual signalling due to their fitness trade‐offs.

Thus, Zahavi's handicap hypothesis is not supported by Grafen's models or any other theoretical models, and rejecting the Handicap Paradigm has important implications for interpreting the past three decades of theoretical and empirical studies (Maynard Smith & Harper, 2003; Searcy & Nowicki, 2005), as well as future research on honest signalling. The Handicap Paradigm needs to be replaced with a Darwinian framework for explaining honest and dishonest signals (Fig. 2). We need to determine how fitness costs and benefits (and their trade‐offs) influence the evolution of honest (and dishonest) signals. We also need to find ways to test whether condition‐dependence of signalling is due to adaptive phenotypic plasticity *versus* inescapable, physical constraints (index signals). Summarizing more promising approaches to studying honest signals is beyond the scope of this review, but before leaving this topic, we address one final perplexing question: How was Zahavi's handicap hypothesis transformed from ‘laughable nonsense’ into an established scientific principle and ‘the central explanation underlying all forms of animal communication’ (Pomiankowski & Iwasa, 1998) (p. 928)?

## VIII. THE HANDICAP PARADIGM: WHY WAS IT ACCEPTED?

*What is striking about Amotz Zahavi*'*s handicap principle (1975) is that its acceptance as a biological phenomenon appears to have been driven by the history of its mathematical modelling* (Grose, 2011, p. 678).

Grafen's (1990a,*b*) publications were key to the acceptance of the Handicap Principle (Fig. 1), but why were his arguments so widely accepted and why do they remain popular? There have been an enormous number of studies on honest signalling, and yet there is still no unequivocal empirical evidence for the Handicap Principle or Grafen's models (Searcy & Nowicki, 2005; Számadó & Penn, 2015). Why have Grafen's theoretical models carried so much weight (Grose, 2011)?

There are several non‐exclusive explanations for the acceptance of the Handicap Principle and Grafen's handicap conclusions. First, the widespread acceptance of Grafen's ‘brilliant but arcane mathematical analysis of biological signals’ (Getty, 2006, p. 83) is often attributed to the overwhelming support it received by respected authorities in the field (Grose, 2011). John Maynard Smith concluded that Grafen's models support Zahavi's general thesis for the evolution of signalling, and his specific argument about sexual selection (Maynard Smith, 1991). He gave a public apology to Zahavi for his scepticism at a meeting of the Royal Society and declared that he had been mistaken (Harper, 2006). Richard Dawkins revised his scepticism after reading Grafen's unpublished manuscripts. In his second edition of *The Selfish Gene*, he stated: ‘If Grafen is correct – and I think he is – it is a result of considerable importance for the whole study of animal signals. It might even necessitate a radical change in our entire outlook on the evolution of behaviour… The Zahavi–Grafen theory, if true, will turn topsy‐turvy biologists' ideas of relations between rivals of the same sex, between parents and offspring, between enemies of different species…it means that theories of almost limitless craziness can no longer be ruled out on commonsense grounds’ (Dawkins, 1989, p. 313). Hamilton (2001) revised his views and suggested that Grafen should receive a good portion of the credit for the ‘Zahavi–Grafen Principle’ because he clarified Zahavi's ‘extremely vague’ and ‘confused’ papers (p. 211). Grafen's models gained even more prestige after Michael Spence was awarded a Nobel Prize in economics for his signalling theory model in 2001, and theoreticians concluded that Grafen had placed the Handicap Principle on a ‘firm mathematical footing’ (Hammerstein & Hagen, 2005). Grafen himself continued to gain status due to his other important theoretical contributions in evolutionary biology. The endorsements by these and other eminent scientists gave legitimacy to the Handicap Principle, which appears to have generated a bandwagon effect. Ironically, Veblen (1899) was among the first to propose that erroneous ideas in human societies spread by emulating high‐status individuals (i.e. bandwagon effects) (Leibenstein, 1950). Thus, Grafen managed to persuade highly influential authorities, including Zahavi's most outspoken critics, but why were they persuaded and why were their arguments so influential?

Second, as we showed, Zahavi's (1977b) condition‐dependent signalling hypothesis was already confused with the handicap hypothesis well before Grafen (1990a,*b*) declared that they are equivalent concepts and provided theoretical support for this hypothesis. Grafen thus embraced a misconception that was already widely assumed, which helps explain why his conclusions were initially accepted. It is unclear why this confusion has not previously been pointed out, at least to our knowledge; neither by supporters who emphasize the theoretical plausibility of Grafen's model, nor critics who have stressed its limitations (Getty, 1998a,*b*, 2006; Számadó, 2000). The muddling of Zahavi's two different proposals for honest signalling was crucial to the initial and continued acceptance of the Handicap Principle, but we do not want to give the impression that this confusion is the complete explanation.

Third, it has been suggested that too much weight has been placed on theoretical models over empirical results (Grose, 2011). After all, as Grafen (1987) pointed out, ‘Danger lies in taking theoretical discussions too literally’ (p. 224). We agree that unambiguous evidence for the Handicap Principle is lacking, but absence of evidence is not evidence of absence. Rejecting a hypothesis based on a lack of evidence is always problematic, and previous reviewers complained that this matter has not been settled because there have been too few attempts to empirically test the Handicap Principle correctly (Kotiaho, 2001; Searcy & Nowicki, 2005). Like many, these reviewers assumed that the correct interpretation of the Handicap Principle is Grafen's strategic choice model. Empirical tests of this model are rare, but they do not provide tests of the Handicap Principle. Because the Handicap Principle is widely assumed to be theoretically established, researchers have instead focused on testing Zahavi's predications of the handicap hypothesis, such as measuring the absolute costs of signals [however, most studies have only measured only energetic, immunological and other proximate mechanisms, and assumed that they provide reliable proxies for viability costs (Kotiaho, 2001)]. The problem is that there are many ambiguous results from empirical studies that have been interpreted as confirming the Handicap Principle, and anomalies – such as costly deception – have too often been ignored. Our theories influence what we observe, the empirical data that we collect, and how we interpret our results. Thus, the question is, why has there been such a strong confirmatory bias for the Handicap Principle? This confirmatory bias includes theoretical as well as empirical results. As we showed, theoretical models showing that signal costs are unnecessary to maintain honesty have been interpreted as ‘handicap models’ and ‘costly signalling theory’ (Godfray, 1991; Godfray & Johnstone, 2000). Theoretical studies showing that signal costs at the equilibrium are not necessary or sufficient to explain the evolution of honest signals (Hurd, 1995; Getty, 1998a; Számadó, 1999; Lachmann *et al*., 2001) have been ignored for too long. Thus, the problem is not that theoretical models have been given too much weight, but rather there has not been enough attention paid to theoretical models and empirical results that reach anomalous conclusions.

Finally, the acceptance of Grafen's handicap conclusions appears to be the result of a logical mistake called *affirming the consequent* (T. Getty, personal communication), and this fallacy may explain most, if not all of the problems described above. This is a fallacy due to taking a true conditional statement and invalidly inferring its converse, as in the following example: ‘If an animal is a dog, then it has four legs. My cat has four legs, and therefore, my cat is a dog’. Similarly, the following logic seems to have been applied to Grafen's model: ‘If a signalling model is a handicap model, then it aims to explain honesty with signal costs. Grafen's signalling model involves costs and aims to provide a general explanation for honesty, and therefore it is a handicap model’. Such reasoning is often used for arguments *aspiring* to be deductively valid, but it does not guarantee that the conclusion is necessarily correct. Empirical results that have been interpreted as supporting the Handicap Principle often appear to have committed the fallacy of affirming the consequent: ‘Handicaps provide honest signals of quality, and therefore, if we find a character that is costly and it honestly signals quality, then it must be a handicap’.

## IX. CONCLUSIONS

*The handicap principle has had an important role in stimulating theory development and empirical research, but it has outlived its usefulness and become an impediment to progress. It is time to usher the handicap principle off to an honourable retirement* (Getty, 2006, p. 87).

**Figure 3:**
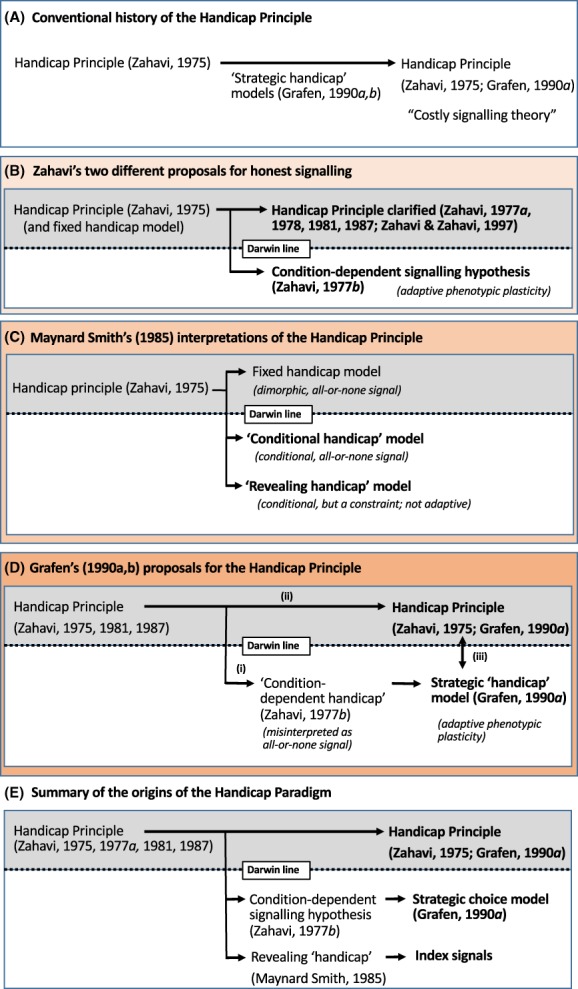
Different perspectives of the theoretical development of the Handicap Principle. (A) The conventional view is that Zahavi's (1975) handicap hypothesis and his broader claims for the Handicap Principle were validated by Grafen's (1990a) ‘handicap’ model. (B) After Zahavi's (1975) original proposal was criticized, he clarified his arguments for his Handicap Principle, which all defy Darwinian logic (dark shading). At the same time, Zahavi (1977b) also proposed another hypothesis (condition‐dependent signalling) that is logical and consistent with evolutionary biology (no shading). The ‘Darwin line’ emphasizes this crucial distinction between these proposals, which has not been previously recognized. (C) Maynard Smith (1985) reviewed three different models of good‐genes sexual selection, which he assumed were all models of the Handicap Principle. These were based on different proximate mechanisms and functions (fixed *versus* plastic expression and constraint *versus* functional), and he overlooked that two of these models were logical and Darwinian (light shading). (D) Grafen (1990a) provided a model of (*i*) Zahavi's (1977b) condition‐dependent ‘handicap’ hypothesis, and he concluded that his model; (*ii*) validates; and (*iii*) is equivalent to Zahavi's (1975, 1981, 1987) Handicap Principle. (E) An historical overview reveals how the Handicap Principle became a confusing mixture of Zahavi's handicap proposals, which are contrary to Darwinian logic (dark shading), and other ideas that are logical and consistent with evolutionary biology (no shading).

1. Zahavi's Handicap Principle has been the leading explanation for the evolution of honest signals in the biological sciences since it was reportedly validated by Grafen's (1990a,*b*) theoretical models (Fig. 3A). It has subsequently been shown that signal costs paid at the equilibrium are neither necessary nor sufficient to explain the evolution of signal reliability at the evolutionary equilibrium, although these findings are not as well known. The Handicap Principle remains popular, but as there is little agreement over how to define or test this idea, research on honest signalling has become bogged down in a quagmire. To understand this problem better, we conducted a comprehensive review of the Handicap Principle and its theoretical development. We show how the Handicap Principle differs from Grafen's model and how these ideas nevertheless became confused with each other.
2. Zahavi (1975) originally aimed to explain the evolution of secondary sexual signals, which he described as ‘handicaps’ to survival (Fig. 3B). He argued that such traits evolve because of and not despite their viability costs, and that they have extra costs that (somehow) ensure their reliability. He also proposed a verbal (fixed handicap) model to explain how his hypothesis might work, but his arguments and his model were both rejected. Zahavi attempted to clarify his hypothesis, and he argued that signals are wasteful and it is their wastefulness that maintains honesty. Moreover, he argued that the handicap hypothesis provides a general principle that explains all types of signalling systems (i.e. the *Handicap Principle*) (Zahavi, 1977a, 1981, 1987).
3. Zahavi (1977b) proposed a second hypothesis to explain honest signals **(**Fig. 3B): he argued that secondary sexual signals are reliable indicators of quality because males are able to adjust signal expression adaptively, according to their quality, and that low‐quality males pay higher viability costs for signalling compared to high‐quality males. His condition‐dependent signalling hypothesis was further developed by Nur & Hasson (1984), who showed that it is logical and consistent with Darwinian principles. Zahavi's hypothesis was given many different labels and interpretations, however, it was confused with the Handicap Principle.
4. Maynard Smith (1985) proposed that there are three types of handicap models (Fig. 3C); however, he misinterpreted Zahavi's (1977b) condition‐dependent signalling hypothesis, and he mistakenly equated this and other putative handicap models to good‐genes sexual selection. These models were rejected by some, and although others suggested that one might sometimes work (the so‐called *revealing handicap)*, it was mistakenly assumed to be a handicap model.
5. Grafen (1990a,*b*) argued that his models vindicate Zahavi's Handicap Principle (Fig. 3D), but this conclusion was based on several misinterpretations. His strategic choice model provided support for Zahavi's (1977b) condition‐dependent signalling hypothesis, but not the Handicap Principle. Honest signals are not wasteful in Grafen's model, and they evolve despite and not because of their trade‐offs on viability, and thus it provides a Darwinian *alternative* to the Handicap Principle. Critics have emphasized the limitations of Grafen's model for explaining the evolution of honest signalling (Fig. 2), but overlooked that it is not a model of the handicap hypothesis.
6. The Handicap Principle was accepted based on misinterpretations of Grafen's models, and there are several reasons why his conclusions were accepted: (*i*) Grafen's conclusions were accepted by influential authorities, which resulted in a scientific bandwagon; (*ii*) His strategic choice model supported Zahavi's (1977b) second signalling hypothesis, which was already confused and equated with the Handicap Principle; (*iii*) Continued acceptance appears to be due to confirmatory bias for both theoretical and empirical results and contrary evidence has too often been ignored; and (*iv*) Grafen's models aim to explain the evolution of honest signals and they involve signal costs, and therefore, it was assumed to be a handicap model (i.e. a logical fallacy known as *affirming the consequent*).
7. The handicap paradigm (and so‐called ‘costly signalling theory’) should be abandoned and replaced with a Darwinian framework (Fig. 3E). Much work is needed to reinterpret three decades of theoretical and empirical studies and to clarify better how fitness costs and benefits (and cost/benefit trade‐offs) influence the evolution of reliable (and dishonest) signals. Models are needed, for example, to distinguish between signals that are honest due to adaptive phenotypic plasticity *versus* actual constraints (indices), and that do not confuse either explanation with the Handicap Principle.
